# Linking *Pedobacter lusitanus* NL19 volatile exometabolome with growth medium composition: what can we learn using comprehensive two-dimensional gas chromatography coupled to time-of-flight mass spectrometry?

**DOI:** 10.1007/s00216-022-04505-6

**Published:** 2023-01-11

**Authors:** Gonçalo Figueiredo, Carina Pedrosa Costa, Joana Lourenço, Tânia Caetano, Sílvia M. Rocha, Sónia Mendo

**Affiliations:** 1 Department of Biology & CESAM, University of Aveiro, Campus Universitário de Santiago, 3810-193 Aveiro, Portugal; 2 Department of Chemistry & LAQV-REQUIMTE, University of Aveiro, Campus Universitário de Santiago, 3810-193 Aveiro, Portugal

**Keywords:** *Pedobacter lusitanus* NL19, *Pedobacter himalayensis* MTCC6384, Volatilome, Microbial metabolomics, GC × GC-ToFMS, HS-SPME

## Abstract

Microbial metabolomics allows understanding and to comprehensively analyse metabolites, and their related cellular and metabolic processes, that are produced and released to the extracellular environment under specific conditions. In that regard, the main objective of this research is to understand the impact of culture media changes in the metabolic profile of *Pedobacter lusitanus* NL19 (NL19) and *Pedobacter himalayensis* MTCC 6384 (MTCC6384) and respective influence on the production of biotechnologically relevant compounds. Solid-phase microextraction combined with comprehensive two-dimensional gas chromatography coupled to time-of-flight mass spectrometry with time-of-flight analyser (GC × GC-ToFMS) was applied to comprehensively study the metabolites produced by NL19 and MTCC6384 both in tryptic soy broth 100% (TSB100) and tryptic soy broth with 25% casein peptone (PC25). A total of 320 metabolites were putatively identified, which belong to different chemical families: alcohols, aldehydes, esters, ethers, hydrocarbons, ketones, nitrogen compounds, sulphur compounds, monoterpenes, and sesquiterpenes. Metabolites that were statistically different from the control (sterile medium) were selected allowing for the construction of the metabolic profile of both strains. A set of 80 metabolites was tentatively associated to the metabolic pathways such as the metabolism of fatty acids, branched-chain aminoacids, phenylalanine, methionine, aromatic compounds, and monoterpene and sesquiterpene biosynthesis. This study allowed to better understand how slight changes of the culture media and thus the composition of nutrients impair the metabolic profile of bacteria, which may be further explored for metabolomics pipeline construction or biotechnological applications.

**Supplementary Information:**

The online version contains supplementary material available at 10.1007/s00216-022-04505-6.

## Introduction

Biochemical processes that take place within a bacterial cell give rise to a complex mixture of metabolites that reflect the conditions under which microorganisms grow. As such, parameters such as culture medium composition, pH, temperature, among others, represent the first line of response of a microorganism to conditions it encounters. Logically, all these parameters influence the microbial metabolome, and its study will be fundamental for the identification of metabolic and cellular pathways of specific compounds. Under each abovementioned specific conditions, a set of metabolites are released to the extracellular environment, the exometabolome, which mirrors the catabolic, metabolic, and anabolic reactions that occur in a given organism [1, 2]. Furthermore, the exometabolome allows to understand strain/species-level differentiation in distinct sets and the richness of intra- and inter-kingdom interactions at several levels [2]. Microbial volatile organic compounds (MVOCs) in particular have been gaining relevance due to the implementation of improved analytical methods and data analysis techniques. Some studies have shown that there are species-level differences in MVOC production between pathogenic and commensal microbial species, suggesting the need for further studies [3]. Consequently, microbial volatilomics has proven to be a powerful tool and has contributed to demystifying microbial biological systems [3, 4]. It is a constantly evolving comprehensive approach that finds application in many sectors, from ecological research to clinic [1, 5]. MVOCs are small molecules (< 300 Da) belonging to several chemical classes, which can evaporate at room temperature and diffuse through air [6]. Microbial metabolomics seems to be a powerful approach to distinguish different cellular states due to growth phase, temperature, oxygen, pH, and nutrient availability [2, 7]. One-dimensional gas chromatography (1D-GC) may be employed in microbial metabolomics; however, metabolomics samples are complex and may require more powerful separation methods such as comprehensive two-dimensional gas chromatography coupled to mass spectrometry with a time-of-flight analyser (GC × GC-ToFMS). The latter is based on the use of two orthogonal mechanisms to separate samples by employing two columns coated with different stationary phases. Furthermore, GC × GC-ToFMS increases sensitivity and improves detection limits, making this technique a great approach to unveil new microbial metabolic profiles [8]. This methodology has been applied with great success to study volatilomes [5].

Our group has been working on secondary metabolites (SM) of a new bacterial species, *Pedobacter lusitanus* NL19 (NL19) from the *Pedobacter* genus. In selected growth conditions (low concentration of casein peptone, 25%-PC25), NL19 produces compounds with antimicrobial activity against Gram-positive and Gram-negative bacteria and yeast. Nonetheless, in culture media with high concentrations of casein peptone (TSB100), antimicrobial activity was completely abolished. Further, this strain presents a relevant biotechnological profile, as it encodes in its genome other secondary metabolites with foreseen application in the most diverse sectors, including human health [9]. NL19 is phylogenetically related to *Pedobacter himalayensis* MTCC6384 (MTCC6384), which our group also concluded to be a producer of antimicrobial compounds with different nature and spectra of activity of those produced by NL19 [9–11]. In addition, it also encloses in its genome a promising and unusual repertoire of biotechnologically relevant SM [10]. Therefore, we were interested in investigating and understanding the intricate metabolic interference of growth media composition on secondary metabolites produced by both strains once big differences are observed when these two strains are grown in TSB100 or PC25.

The main objective of this research was to understand the impact of culture media composition on the alteration of volatile exometabolome, by comparing differences detected in the metabolic profile of each species, when cultivated in two different culture media (TSB100 and PC25) which are known to affect the production of bioactive compounds, like pedopeptins [9]. This study constitutes the basis for future studies where knowledge of the impact of culture media on the production of secondary metabolites with biotechnological application, namely, clinical setting, food industry, veterinary, aquaculture, among others, is fundamental. Strains were cultivated in each of the culture media until the lag phase, approximately (48 h). The exometabolome was analysed through headspace solid-phase microextraction (HS-SPME) combined with GC × GC-ToFMS. After, hierarchical clustering analysis, plot comparisons, and statistical analysis allowed to compare differences between each strain and between different media, contributing to unveil their metabolic profiles. Finally, identified metabolites were associated to putative metabolic pathways.

## Materials and methods

### Microorganisms and growth conditions

NL19 and MTCC6384 were grown on tryptic soy agar (TSA) plates for 48 h at 26 °C [12]. The following growth media were used: TSB100 (TSB: casein peptone 15 g/L, soy peptone 5 g/L, and NaCl 5 g/L) and PC25 (PC25: casein peptone 3.75 g/L, soy peptone 5 g/L, and NaCl 5 g/L). For each species, a pre-inoculum was prepared in 50-mL falcon tubes containing 20 mL of TSB100. For each strain, 2–3 individual colonies were inoculated in 20 mL of TSB and incubated overnight at 26 °C and 180 rpm. This pre-inoculum was used to inoculate the test shake flasks. Two conditions were tested — TSB100 and PC25. Each strain was suspended in 25 mL of TSB100 and PC25 to a final concentration of OD_600nm_ = 0.05, in 100-mL shake flasks. Control samples consisted of the same volume of cell-free growth medium. Three replicates of each test condition and control were prepared. Shake flasks were incubated in an orbital shaker for 48 h, at 26 °C and 180 rpm. The following conditions were analysed: NL19 in TSB100 (PL100), NL19 in PC25 (PL25), MTCC6384 in TSB100 (PH100), MTCC6384 in PC25 (PH25%), control TSB100 (TSB100), and control PC25 (PC25).

After incubation, the number of colony-forming units per millilitre (CFU/mL) was determined as follows: 0.1 mL was withdrawn from each culture and OD_600nm_ was measured. CFU/mL estimation was used to normalize areas of the detected metabolites. CFU/mL in each OD_600nm_ was determined, under the same conditions, and using the single plate-serial dilution spotting (SP-SDS) method [13].

### Determination of the volatile exometabolome

#### Sample preparation for headspace analysis

After incubation, cultures were collected into 50-mL falcon tubes. To quench the samples, all tubes were centrifuged at 13,000 rpm for 15 min. Supernatants were quickly separated from the pellet into new falcons and were filtered with the help of a vacuum filtration system equipped with a GN-6 Metricel® Grid 47-mm filter mixed cellulose esters with 0.45-µm pore size, to remove remaining cells and other compounds that may compromise the analysis. After filtration, 18 mL of each filtrate was transferred to 60-mL glass vials containing 4 g of NaCl and a stirring bar of 20 × 5 mm. The vial was closed with a silicone/polytetrafluoroethylene septum and aluminium cap and stored at − 80 °C until analysis [14].

#### Headspace analysis by GC × GC-ToFMS

The headspace analysis was performed by HS-SPME/GC × GC-ToFMS methodology, as described by Costa et al., 2016 [14]. The SPME fibre used included a fused silica fiber coating, partially cross-linked with 50/30 µm divinylbenzene/carboxen™/polydimethylsiloxane StableFlex™ (1 cm) (Supelco, Aldrich, Bellefonte, PA, USA), which presents a wide range of sorbing capacity for compounds with distinct physicochemical properties. The defrosted vials undergo extraction in a thermostated water bath at 50 °C, for 30 min, under agitation (350 rpm). Then, the SPME fibre was manually inserted into the injection port (250 °C) of the equipment GC × GC-ToFMS LECO Pegasus 4D (LECO, St. Joseph, MI, USA). The inlet was lined with a 0.75-mm I.D. glass liner and splitless injection mode was used (30 s). The GC × GC-ToFMS system comprises an Agilent GC 7890A gas chromatograph (Agilent Technologies, Inc., Wilmington, DE), with a dual stage jet cryogenic modulator (licensed from Zoex) and a secondary oven. An Equity-5 column (5% diphenyl/95% dimethyl siloxane, 30 m × 0.32 mm I.D., 0.25-µm film thickness, Supelco, Inc., Bellefonte, PA, USA) and a DB-FFAP column (nitroterephthalic-acid-modified polyethylene glycol, 0.79 m × 0.25 mm I.D., 0.25-µm film thickness, J&W Scientific Inc., Folsom, CA, USA) were used for first and second dimensions, respectively. Helium was used as the carrier gas at a constant flow rate of 2.50 mL min^−1^. The following temperature programmes were used: the primary oven temperature was ranged from 40 °C (1 min) to 145 °C at 5 °C min^−1^, and then to 200 °C (1 min) at 7 °C min^−1^. The secondary oven temperature programme was 5 °C offset above the primary oven. Both the MS transfer line and MS source temperatures were 250 °C. The modulation period was 5 s, keeping the modulator at 20 °C offset above the primary oven, with hot and cold pulses by periods of 0.80 and 1.70 s, respectively. The ToF analyser was operated at a spectrum storage rate of 100 spectra s^−1^, with a mass spectrometer running in the EI mode at 70 eV and detector voltage of − 1523 V, using an *m*/*z* range of 35–300. Automated data processing software ChromaTOF® (LECO) was used to process total ion chromatograms, at signal-to-noise threshold of 100. For identification purposes, the mass spectrum and retention times of the analytes were compared with standards, when available. The mass spectrum of each peak was compared to those existing in mass spectral libraries, including an in-house library of standards and two commercial databases (Wiley 275 and US National Institute of Science and Technology (NIST) V.2.0–Mainlib and Replib). Moreover, a manual analysis of mass spectra was performed using additional information such as linear retention index (RI) value, experimentally obtained through van Den Dool and Kratz RI equation [15]. To determine the RI, a C_8_-C_20_ *n*-alkanes series was used (solvent *n*-hexane was used as C_6_ standard), comparing these values with reported ones in existing literature for chromatographic columns similar to first dimension column. The RI parameter calculated also supports the identification, since the calculated retention index (RI_Calc_) differed 0 to 5.8% when compared to literature data (RI_Lit_) for the ^1^D column or equivalents, corresponding to a median of 0.4% and a standard deviation of 0.8%. The majority (> 90%) of compounds show a variation of ≤ 2%. Also, the majority (> 90%) of identified compounds showed similarity matches > 800/1000. The deconvoluted total ion current GC × GC area data were used as an approach to estimate the relative content of each metabolite of the strains under study.

### Data analysis

Peak areas were extracted from chromatograms and used to form a data set of 18 observations (2 species × 2 different media, each one in triplicate = 12, and 2 controls, each one for each type of medium = 2 media, each one with 3 replicates) and 320 variables (see Electronic Supplementary Information — Table S1). Bubble plots were built which included all identified compounds and raw peak areas to take a quick snapshot of the headspace composition of each sample. Further analysis of the number of compounds and respective chemical families was also carried out. MetaboAnalyst 5.0 (web software, The Metabolomics Innovation Centre (TMIC), Edmonton, AB, Canada) software was used to produce a hierarchical clustering analysis with a heatmap that allowed to compare the abundance of the 320 variables in each condition, with and without normalization by probabilistic quotient normalization (PQN) and CFU/mL. Autoscaling was applied to each variable. A dendrogram was also made to analyse relational distance quantitatively. In addition, normalized data (CFU per condition) is also provided as a bar plot.

The significance of the analytes was compared to assess which of the 320 variables were statistically significant. All test conditions were compared with the respective control (PL100-TSB100, PL25-PC25, PH100-TSB100, and PH25-PC25). Analytes that presented a *p*-value of < 0.05 were considered significant, and thus were selected through the application of an ANOVA or Kruskal–Wallis test with post hoc analysis with Tukey test or Dunn test with Holm correction (R software™ v.4.1.2 — R Foundation for Statistical Computing, Vienna, Austria). In total, 123 variables were selected, after previous statistical analysis, and these are marked in bold in Online Resources 1 and 2. Variation in the relative abundance of each compound is represented by an arrow, where arrows pointing up or down represent high or low abundance, respectively, when compared to control (see Electronic Supplementary Information — Table S2). Selected metabolites were normalized with the respective CFU estimation for each condition, which allowed a comparison between each bacterium and both growth media. An additional heatmap was constructed using data from the 123 variables, by applying CFU normalization and autoscaling.

To assess the effect of the medium in each bacterium growth (PL100-PL25 and PH100-PH25), an ANOVA was performed with multicomparison Tukey test or Kruskal–Wallis with Dunn test using the Holm correction (R software™ v.4.1.2), with a significance level of 0.05. *p*-values of the 24 analytes, that were considered significant (*p* < 0.05), are marked in bold. Arrows indicate the condition with a higher abundance of a given analyte. Statistically different values for each strain were used to produce bubble plots containing normalized peak area, which allowed the comparison of abundances of each analyte between the two-growth media.

To understand the role of the 123 selected metabolites, a network of metabolic pathways was designed using information available in the databases (KEGG, Kanehisa Laboratories, Kyoto, Japan; MBRole 2.0, Spanish National Centre for Biotechnology, Madrid, Spain) and also in the literature. This exercise allowed the association of each metabolite to a putative metabolic pathway, as well as the possible contribution of each identified pathway. Each metabolite was associated with the heatmap for each condition, which allowed us to quickly understand variations between conditions. Statistically irrelevant metabolites, when compared to control conditions, were marked with an asterisk (*).

## Results

### NL19 and MTCC6384 volatile exometabolome composition

Electronic Supplementary Information — Table S1 lists the 320 compounds putatively identified in the volatile exometabolomes of PL100, PH100, PL25, and PH25, which also includes detailed information of these metabolites, namely, ID number, first dimension (^1^*t*_R_) and second dimension (^2^*t*_R_) retention times, compound name, formula, CAS number, calculated RI (RI_Calc_), and RI presented in the literature (RI_Lit_). These metabolites were distributed over 11 chemical families: 51 alcohols (15.9%), 24 aldehydes (7.5%), 50 esters (15.6%), 11 ethers (3.4%), 44 hydrocarbons (13.8%), 57 ketones (17.8%), 17 N-compounds (5.3%), 10 S-compounds (3.1%), 44 monoterpenes (13.8%), 9 sesquiterpenes (2.8%), and 3 norisoprenes (0.9%). The respective GC × GC total ion chromatograms can be consulted in Electronic Supplementary Information — Fig. S1.

**Fig. 1:**
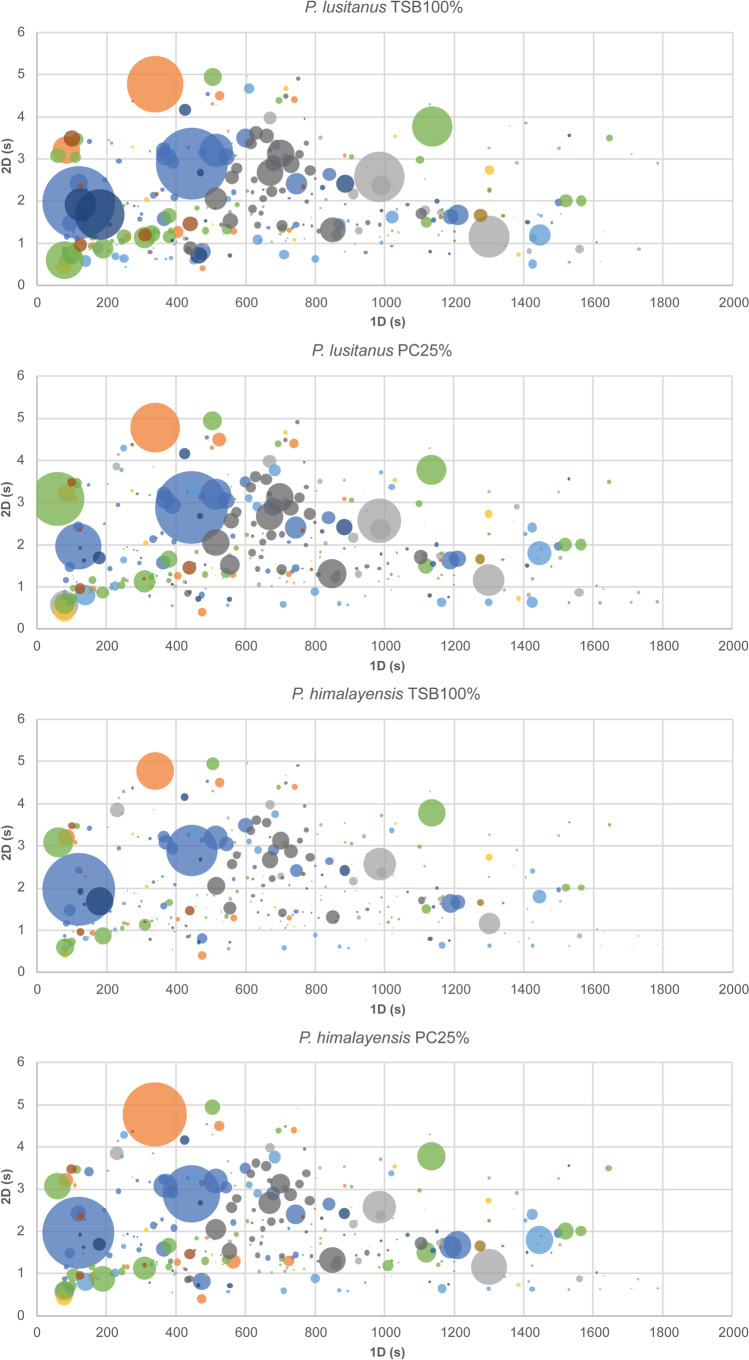
Bubble plot acquired using raw data obtained through GC × GC-ToFMS analysis representing each condition assessed — *P. lusitanus* TSB100% (PL100), *P. lusitanus* PC25% (PL25), *P. himalayensis* TSB100% (PH100), and *P. himalayensis* PC25% (PH25). The first dimension of the retention time in seconds (^1^D) is plotted in the *X*-axis and the second dimension of the retention time in seconds (^2^D) is plotted in the *Y*-axis. Each colour is associated with a specific chemical family:  Alcohols,  Aldehydes,  Esters,  Ethers,  Hydrocarbons,  Ketones,  N-compounds,  S-compounds,  Terpenes  Sesquiterpenes, and  Norisoprenes

Bubble plots presented in Fig. 1 contain all the information of the bidimensional retention times and respective abundance translated into bubble size. Furthermore, in all the conditions analysed, the majority of the compounds belong to alcohols (15.9%), esters (15.6%), and ketones (17.8%). Analysis of the bubble plots shows that the metabolic profiles are similar, once raw peak values are used. High peak areas, that indicate a high abundance of compounds, can be observed in PL100 and PL25 when compared to PH100 and PH25. PH100 condition has the lowest peak areas compared to other conditions. It can also be noted that chemical families are well grouped, regarding the ^1^D and ^2^D.

**Fig. 2:**
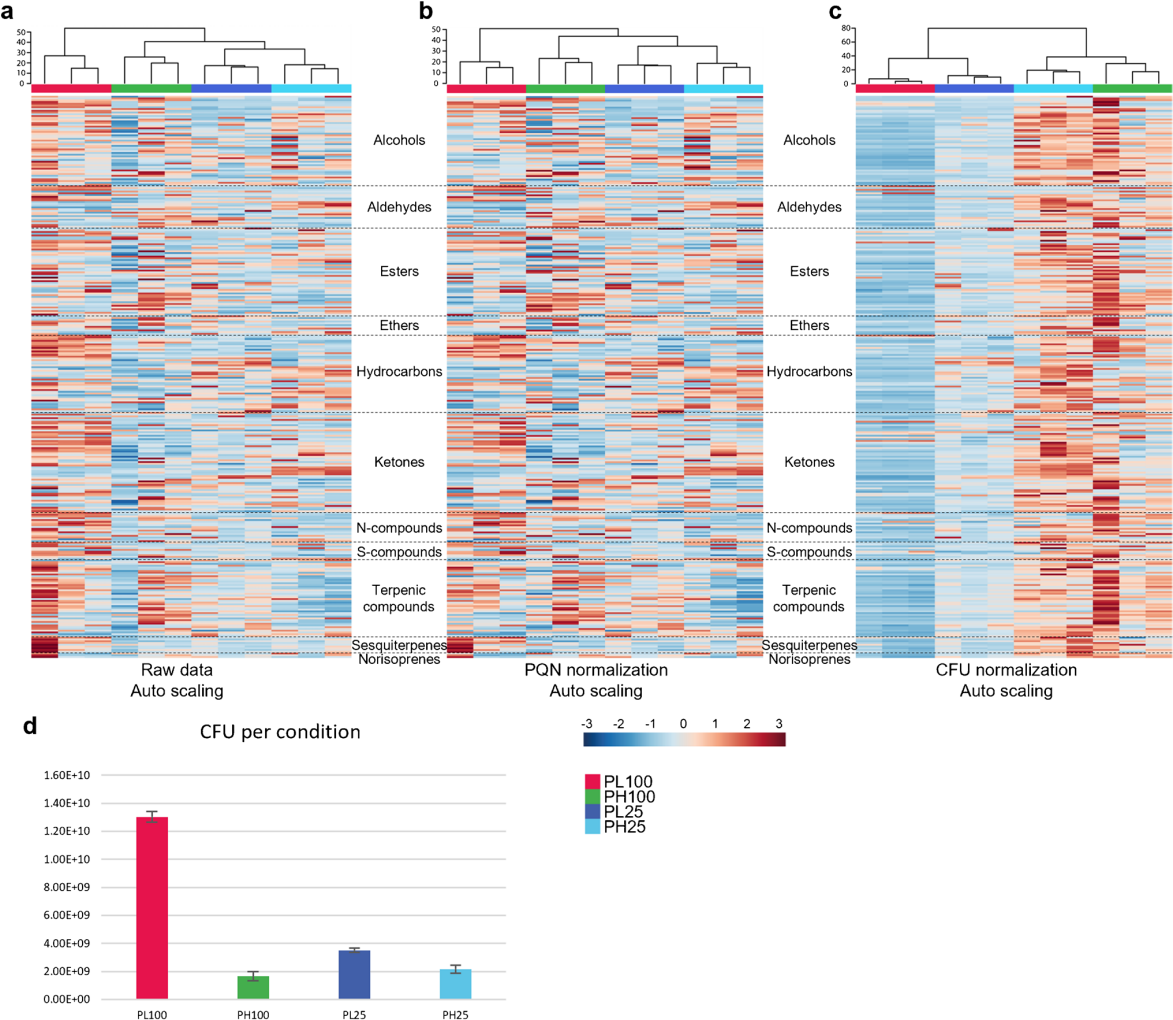
Hierarchical clustering heatmaps (MetaboAnalyst 5.0) visualization of the 320 compounds putatively identified in the headspace of *P. lusitanus* NL19 TSB100% (PL100 — red), *P. himalayensis* MTCC 6384 TSB100% (PH100 — green), *P. lusitanus* NL19 PC25% (PL25 — dark blue), and *P. himalayensis* MTCC 6384 PC25% (PH25 — light blue). Heatmaps differ in that they include (**a**) unnormalized data, (**b**) normalization with probabilistic quotient normalization (PQN), (**c**) normalization with CFU, followed by autoscaling. Each chemical family is separated by dashed lines. Respective hierarchical dendrograms were built at the top of each heatmap using Ward clustering and Euclidean measurement distance. (**d**) The CFU estimate in each condition is plotted in the bar chart. Figure legend with colour code allows identifying respective conditions (red — PL100, green — PH100, dark blue — PL25, and light blue — PH25). Each line represents one compound and each column an independent assay. A chromatic scale from red (maximum) to blue (minimum) is applied, allowing to visually assess the abundance of each putatively identified compound

To better understand the complex data obtained, three hierarchical cluster heatmaps were constructed (Fig. 2), using MetaboAnalyst 5.0, which allows the visualization of the 320 metabolites identified in each condition, represented by a chromatic scale from red (maximum) to blue (minimum), allowing to visually assess the differences of each possible compound. The left heatmap (Fig. 2a) was constructed without data normalization. The heatmap at the centre (Fig. 2b) was built using PQN normalization. Finally, the right heatmap (Fig. 2c) shows data normalized with CFU estimation. The data obtained were treated with autoscaling. Dendrograms with Euclidean measures are shown at the top of each heatmap. CFU estimates for each condition are also plotted (Fig. 2d).

The heatmap without CFU normalization (Fig. 2a) shows that the headspace is both complex and very different between conditions, even when a comparison is made between identical strain and identical cultivation medium. When analysing the dendrogram, it is possible to observe the formation of a cluster containing the conditions PH100, PL25, and PH25. The PL100 condition is isolated, indicating that it is the most different condition. In the abovementioned cluster, the conditions PH25 and PL25 are the closest ones regarding PH100. The isolated cluster from the PL100 condition occurs due to the highest abundance of metabolites in almost all chemical families, compared to the other three conditions, as shown in Fig. 2a. In general, when compared to PC25 medium, TSB100 medium has the highest abundance of metabolites, and this is observed for both strains. The normalization by PQN shows a very similar clustering (illustrated by heatmap and dendrogram, Fig. 2b) to raw data clustering (Fig. 2a). This type of normalization takes in count and explains the dilution of complex mixtures and works with the absolute concentration of each compound. However, when the data is normalized by CFU, we analyse the impact of media at the cellular level, because it can estimate the quantity of MVOCs produced/consumed by each bacterial cell to assess their metabolic state in a given condition. Thus, both normalization methods are complementary; however, the CFU normalization method reveals to be the most appropriate methodology to our study. When the data is normalized by CFU, the dendrograms show a formation of two clusters: one containing PL100 and PL25 conditions and the other with PH100 and PH25 conditions. With the additional information offered by the chromatic scale, it is possible to observe a clear distribution of approximately equal abundances per strain, thus showing the importance of normalization in the analysis of the data, in this case by CFU. Clearly, MTCC6384 has a higher abundance of compounds per CFU for both PH100 and PH25 (Fig. 2c).

### Metabolic profile of NL19 and MTCC6384

A set of 123 of the full data set of 320 compounds were selected (see Electronic Supplementary Information — Table S1 and S2) based on statistical difference between, at least, one of the conditions tested compared to the respective control.

**Fig. 3:**
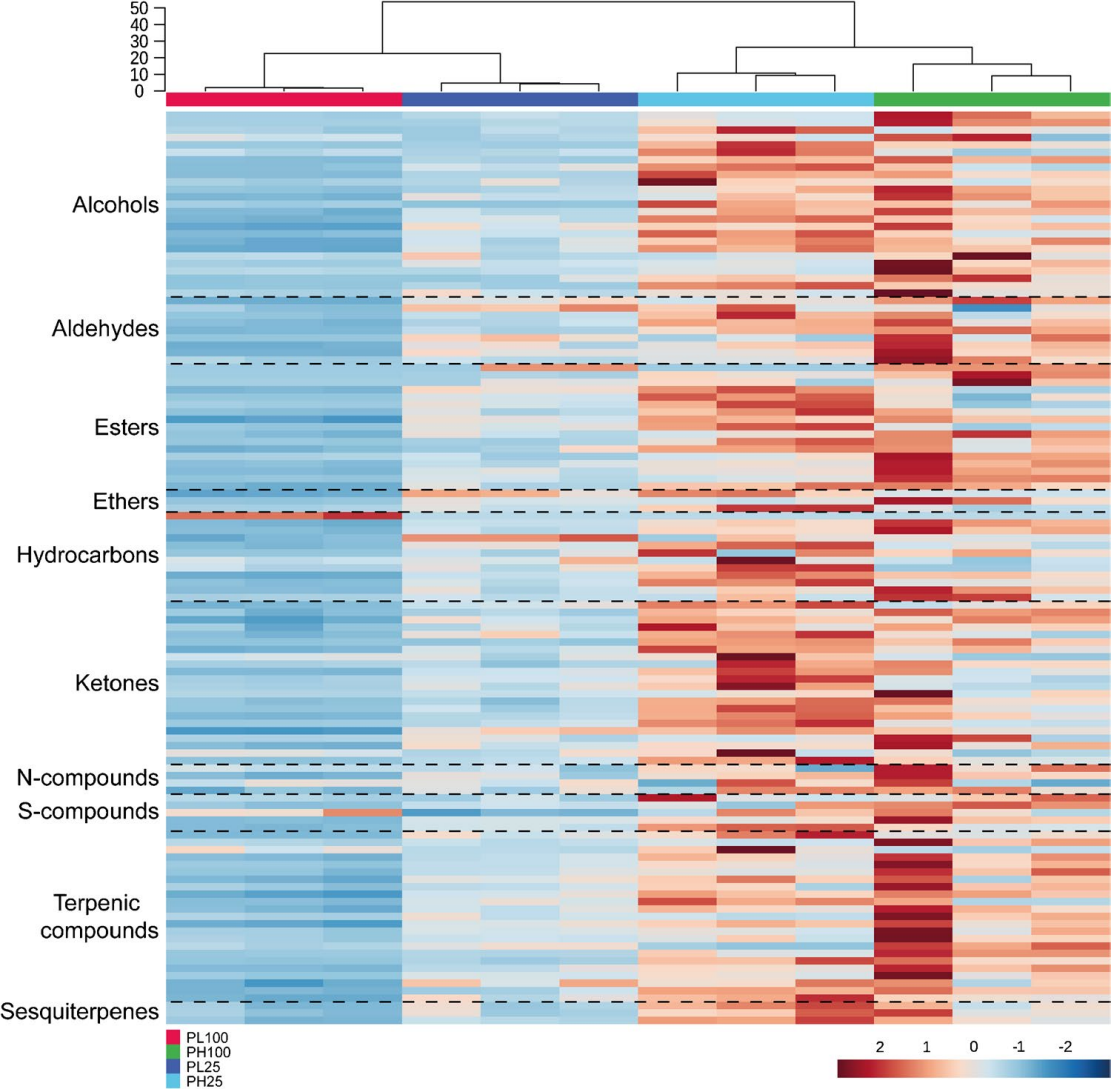
Hierarchical clustered heatmap visualization of the selected 123 compounds with at least one condition that was statistically different (*p* < .05) to the respective control media. The heatmap was built with MetaboAnalyst 5.0 software, using the chromatographic area value with CFU normalization and autoscaling. The compounds are organized by chemical families. Each compound is placed in one individual line and each column represents an independent experiment. The colour code supports the identification of each condition: *P*. *lusitanus* NL19 TSB100% (PL100 — red), *P*. *himalayensis* MTCC 6384 TSB100% (PH100 — green), *P*. *lusitanus* NL19 PC25% (PL25 — dark blue), and *P*. *himalayensis* MTCC 6384 PC25% (PH25 — light blue). A chromatic scale from red (maximum) to blue (minimum) is applied, allowing to visually assess the abundance of each possible identified compound

To understand the impact of the medium on the growth of each species, a hierarchical clustering heatmap was made (Fig. 3), to compare similarities and differences in the presence of each bacterium. Heatmap was built using data normalized with CFU and autoscaling. Two major clusters can be distinguished in the dendrogram: (i) NL19 cluster, which includes both PL100 and PL25 conditions, and (ii) MTCC6384 cluster, including both PH100 and PH25 conditions. The dendrogram shows that the conditions are very similar within the same strain but different conditions. However, they are hugely different between different strains. The metabolites are distributed by chemical families, except for the norisoprenoids family which does not have any metabolite with a statistically significant difference (*p* > 0.05). The formation of two clusters is due to the high abundance of compounds in MTCC6384 and low in NL19. PL100 and PL25 conditions have clades with lower height in relation to PH100 and PH25, indicating a higher similarity between conditions of the NL19 strain. Regarding the chromatic scale, a high content of compounds is observed in PH100 and PH25. This difference contributes to the formation of two clusters, one containing NL19 conditions (PL100 and PL25) and the other containing MTCC6384 conditions (PH100 and PH25). Condition PL25 shows a greater abundance of metabolites belonging to any of the following chemical families: alcohols, aldehydes, esters, ethers, hydrocarbons, ketones, N-compounds, S-compounds, monoterpenes, and sesquiterpenes, whereas in PL100, a lower abundance is observed. Regarding MTCC6384 conditions, a greater variability is observed. In PH100, a higher abundance of alcohols, aldehydes, esters, N-compounds, S-compounds, and monoterpenes is observed, while in PH25 a greater abundance of ethers, hydrocarbons, ketones, and sesquiterpenes is observed. From the 123 selected metabolites, 73 were more abundant and 47 were less abundant in at least one condition when compared to control (sterile TSB100 or PC25 media). Still, pentanal (55), 2-ethoxy-2-methylpropane (126), and nonane (137) are more or less abundant depending on the medium (see Electronic Supplementary Information — Table S2). Most metabolites that appear in statistically higher abundance (*p* < 0.05), regardless of condition, belong to alcohols, hydrocarbons, ketones, N-compounds, monoterpenes, and sesquiterpenes. Conversely, the vast majority of statistically less abundant (*p* < 0.05) metabolites were aldehydes, esters, and S-compounds. The most abundant alcohol in all conditions was 2-ethylhexan-1-ol (28). The least abundant alcohol was α,α,4-trimethylcyclhexanemethanol (49) in PL100 and PH25, 2-methylpentan-1-ol (12) in PH100, and 4-methylpentan-2-ol (9) in PL25. Among the aldehydes, 4-methylbenzaldehyde (71) and deca-2,4-dienal (65) were in high and low abundance, in PH100, PL25, and PH25, respectively, while octanal (60) and pentanal (55) appear in higher and lower abundance, respectively, in PL100. 3-Hydroxy-2,4,4-trimethylpentyl-2-methylpropanoate (101) was the most abundant ester, while methyl methacrylate (77) appears in vestigial (vt) amounts in all four conditions. In addition, isoamyl acetate (81) and 2-methylbutyl acetate (82) were also detected in vestigial abundances in PL100. As for ethers, 2-ethoxy-2-methylpropane (126) was the most abundant in all conditions, while 2-butoxyethanol (127) and 1,1-dimethylethoxybenzene (133) were less abundant in PL100, PH100, PL25, and PL25. In TSB100, 6-methyloct-1-ene (141) is the most abundant hydrocarbon, whereas in PC25 a higher abundance of naphthalene (169) is observed. In all conditions, but PL100, compound nonane (137) remains vestigial. Also, in PL100, the less abundant and vestigial hydrocarbon is 1-ethyl-2-methylbenzene (163). The ketones 5-methylhexan-3-one (191) and 4-methylheptan-2-one (196) are highly abundant in PL100 and PL25, and in PH100 and PH25 conditions, respectively. Still, the less abundant ketones were nonan-3-one (205) in PH100, 1-phenylbutan-1-one (222) in PL100 and PH25, and cyclohexanone (227) in PL25. As for the nitrogen-containing compounds, 4-cyanocyclohexene (242) is highly abundant in all conditions. Yet, 1-isoamylpyrrole (245) is less abundant in PH100, PL25, and PH25 and benzylidenepropylamine (249) in PL100. Among sulphur-containing compounds, methyl thioacetate (255) is highly abundant in PL100, and 2-acetylthiazole (262) in PH100, PL25, and PH25. Conversely, the least abundant sulphur-containing compounds were 3-(methylthio)propanal (259) in PL100, PH100, and PH25, and 2-tertiobutylthiophene (264) in PH100. Levomenthol (isomer) (286) is the most abundant monoterpene in both conditions. α-Pinene (265) appears in trace amounts in PL100, while the least abundant were carveol (294) in PH25 and carvotanacetone (300) in PH100 and PL25. Regarding sesquiterpenes, in all tested conditions, caryophyllene oxide (314) is the most abundant metabolite and δ-cadinene (312) is the least abundant.

### Differences observed for each species in TSB100 and PC25

**Fig. 4:**
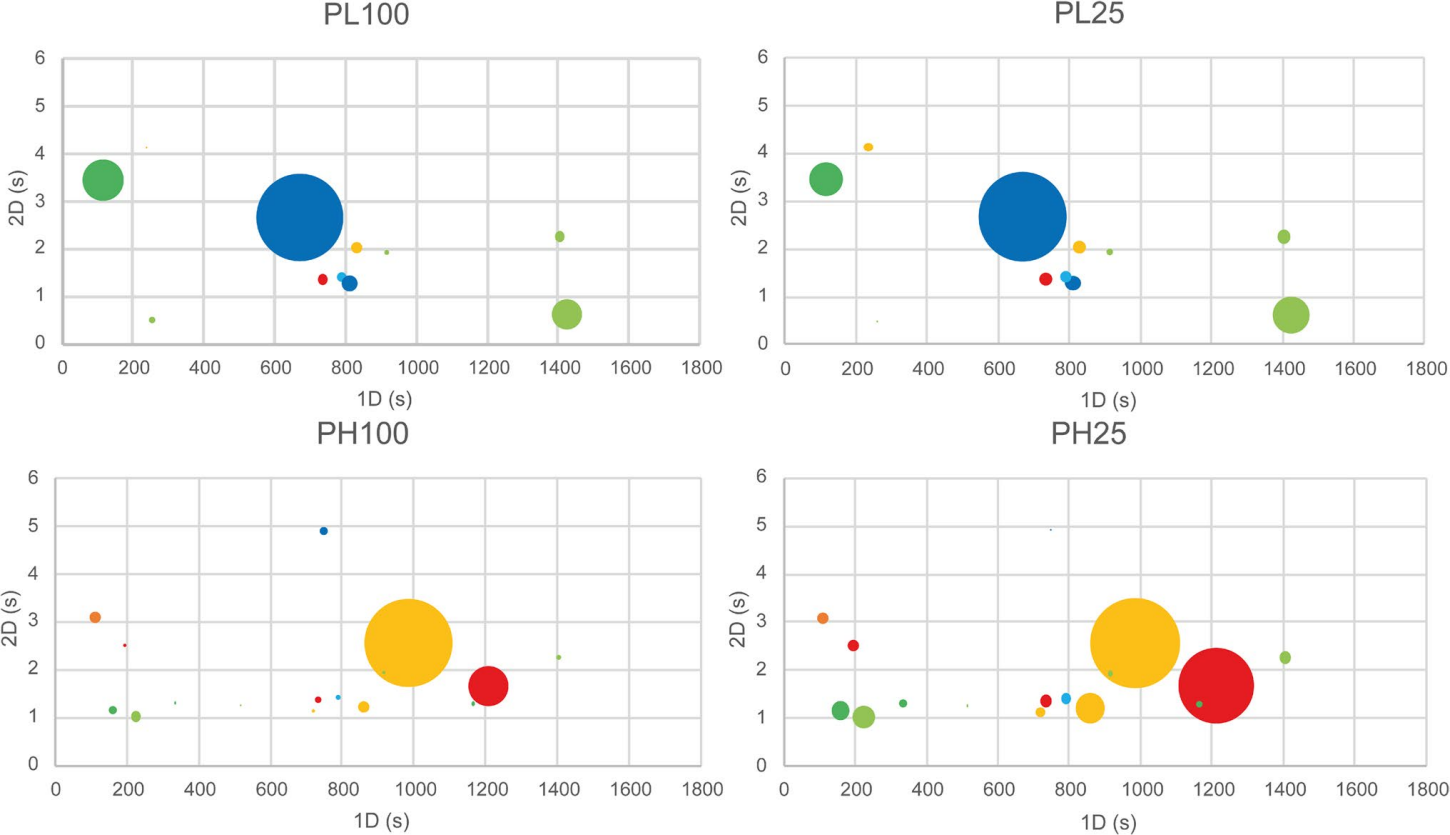
Bubble plots representing the statistically different compounds (*p* < .05) that can differentiate *P. lusitanus* NL19 TSB100% (PL100), *P. himalayensis* MTCC 6384 TSB100% (PH100), *P. lusitanus* NL19 PC25% (PL25), and *P. himalayensis* MTCC 6384 PC25% (PH25), illustrating the *p*-values presented in Table 1. Each bubble is positioned in the same dimensional space (^1^*t*_R_ and ^2^*t*_R_) of the compound that it represents, and the size of the bubble corresponds to the peak area. The colour code identifies each chemical family: red — alcohols; orange — aldehydes; yellow — esters; light green — hydrocarbons; green — ketones; light blue — S-compounds; dark blue — terpenes

A set of 123 metabolites was statistically analysed to compare conditions PL100-PL25 and PH100-PH25. Table 1 contains the 24 metabolites which showed differences in one or both culture media considering that it also stems from the differences between condition and control. These metabolites belong to alcohols, aldehydes, esters, hydrocarbons, ketones, S-compounds, and monoterpenes. Esters and hydrocarbons are the chemical families with the largest number of metabolites showing statistical differences (*p* < 0.05), the majority of them between PH100 and PH25. Despite that, around 83% of the metabolites have a statistical difference (*p* < 0.05) in only one strain (MTCC6384), and 4 metabolites are common to all conditions. Bubble plots (Fig. 4) were built using CFU normalized data to emphasize those differences. Most of the PL100-PL25 statistical differences (*p* < 0.05) are observed in hydrocarbons, esters, and monoterpenes, while for PH100-PH25, the most statistically different families (*p* < 0.05) are esters and hydrocarbons.

**Table 1:** Set of the statistically different compounds (*p* < 0.05) between each media and considering the abundance between the media and the respective control. The *p*-value of these compounds is marked in bold, and it was calculated using an ANOVA and Tukey test or Kruskal–Wallis and Dunn test with Holm correction. The arrow indicates the condition with the highest abundance. The ID and compound name that help their identification are also included

Chemical family	ID	Compound	*P-values*					
			PL100 vs PL25			PH100 vs PH25		
Alcohols	12	2-Methylpentan-1-ol	0.9975			**0.0001**		↑
	37	2-Methyldodecan-2-ol	**0.0380**		↑	**0.0007**		↑
	47	2,4-Di-tert-butylphenol	0.5222			**0.0003**		↑
Aldehydes	55	Pentanal	vt		↑	**0.0019**	↑	
Esters	82	2-Methylbutyl acetate	**vt**		↑	0.0927		
	94	Octyl acrylate	0.3473			**0.0017**		↑
	96	But-1-en-1-yl 4-methylpentanoate	**0.0008**		↑	0.9641		
	97	2-tert-Butylcyclohexyl acetate	0.0396		↑	**0.0002**		↑
	101	3-Hydroxy-2,4,4-trimethylpentyl-2-methylpropanoate	0.0899			**0.0029**	↑	
	119	Benzyl butyrate	0.1076			**0.0001**	↑	
Hydrocarbons	137	Nonane	**vt**	↑		vt -		
	150	Heptadecane	**0.0003**		↑	1.0000		
	160	1,3-Dimethylbenzene	0.0127		↑	**0.0005**		↑
	168	1-Ethyl-2,3-dimethylbenzene	0.7284			**0.0389**		↑
	172	4-Phenylcyclohexene	**0.0262**		↑	**0.0002**		↑
	176	1,1′-(1-Methyl-1,3-propanediyl)bis-benzene	**0.0234**		↑	**0.0017**		↑
Ketones	187	4-Methylpentan-2-one	**0.0243**	↑		0.9974		
	190	4-Methylpent-3-en-2-one	0.0533			**0.0081**		↑
	198	4-Methylhept-3-en-2-one	0.7341			**0.0279**		↑
	216	Tridecan-3-one	0.0001		↑	**0.0479**		↑
S-compounds	264	2-Tertiobutylthiophene	**0.0130**		↑	**0.0002**		↑
Terpenic compounds	286	Levomenthol (isómer)	**0.0029**	↑		0.6133		
	294	Carveol	0.3073			**0.0132**	↑	
	302	Linalyl acetate	**0.0407**	↑		0.9654

For NL19, almost all metabolites are relatively abundant in PL25, except for 137 which is more abundant in PL100. However, regarding the abundance and when considering differences observed in relation to control, the interpretation of the results must be more careful. For example, 2-methyldodecan-2-ol (37) is more abundant in PL25 than in PL100. However, the abundance of this metabolite is lower in both conditions when compared to the control. For MTCC6384, the metabolites seem to be more abundant in PH25, except for 3-hydroxy-2,4,4-trimethylpentyl-2-methylpropanoate (101), benzyl butyrate (119), and carveol (294) which are highly abundant in PH100.

### Putative metabolic pathways

**Fig. 5:**
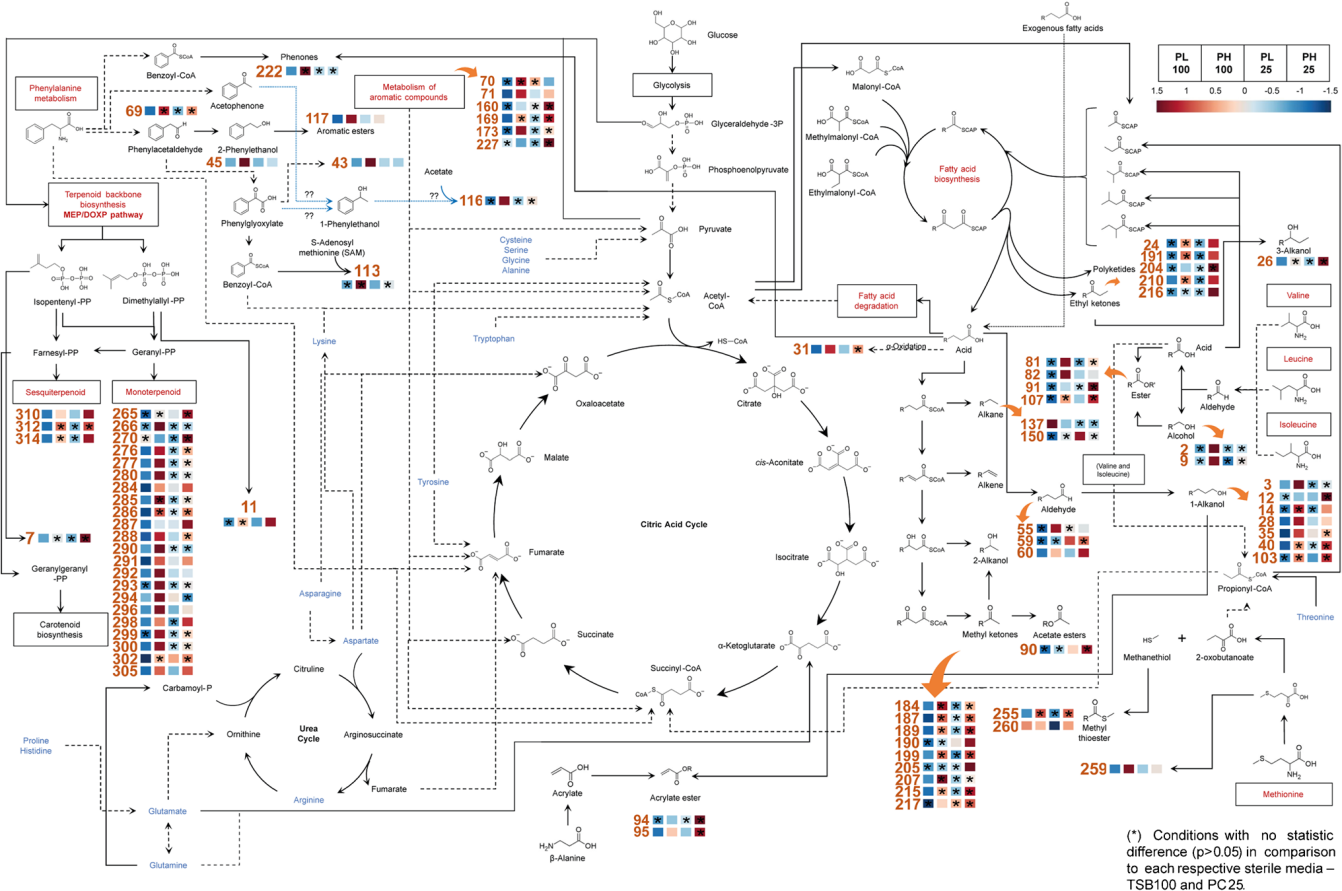
Putative representation proposed for the possible metabolic pathways where certain compounds with statistical difference (*p* < .05) may be involved. Each compound is represented by its ID number followed by the respective heatmap using a colour code to compare each condition (*P. lusitanus* NL19 TSB100% (PL100), *P. himalayensis* MTCC 6384 TSB100% (PH100), *P. lusitanus* NL19 PC25% (PL25), and *P. himalayensis* MTCC 6384 PC25% (PH25)). Each condition is represented by a single-coloured square that results from the group average for each condition. The conditions that do not present statistical significance (*p* > .05) regarding to the respective sterile media are marked with (*). Blue dot marked arrows with ?? indicate hypothetical pathway

Figure 5 illustrates putative metabolic pathways explaining the involvement of 123 selected metabolites, of which only 80 could be associated with putative metabolic pathways. A large number of them are involved in fatty acids and branched-chain aminoacid pathways, resulting from intermediates of these pathways, especially ketones and alcohols. Metabolites oct-1-en-3-ol (24), octan-3-ol (26), 191, 7-methyloctan-3-one (204), decan-3-one (210), and tridecan-3-one (216) result from the synthesis of ethyl ketones. Metabolites nonane (137) and heptadecane (150) result from alkane synthesis from acids that result from the fatty acid pathway. The same occurs for aldehydes (pentanal (55), 2-ethylhexanal (59), octanal (60)), 1-alkanols (butan-1-ol (3), hexan-1-ol (14), 2-ethylhexan-1-ol (28), 5-methylheptan-1-ol (31), nonan-1-ol (35), decan-1-ol (40), ethyl decanoate (103)), acetate esters (3-methylheptyl acetate (90)), and methyl ketones (pentan-2-one (184), 4-methylpentan-2-one (187), hexan-2-one (189), 4-methylpent-3-en-2-one (190), 5-methylheptan-2-one (199), 205, 6-methylhepta-3,5-diene-2-one (207), dodecan-2-one (215), and tridecan-2-one (217)). From this group, statistically different metabolites are observed mainly in PL100 (*p* < 0.05). Significantly different methyl ketones are observed in PL100 and PH25. Significant aldehydes are observed in PH100 and PL25. Four 1-alkanols are significantly different in PL100, PH100, and PL25. 3-Methylheptyl acetate (90), a putative acetate ester from methyl ketones, is significantly different in PL25. Ethyl ketones and 3-alkanols have higher statistically different metabolites (*p* < 0.05) in PH25 and PL100, respectively. Alkanes and 5-methylheptan-1-ol (31) are statistically different (*p* < 0.05) in PL100, PH100, and PL25. Esters isoamyl acetate (81), 2-methylbutyl acetate (82), isononyl acetate (91), and isopropyl laurate (107) may have different origins, with high amount of statistically significant metabolites (*p* < 0.05) in PH100 and PL25. However, alcohols 2-methylpropan-1-ol (2), 4-methylpentan-2-ol (9), and 2-methylpentan-1-ol (12), which arise from branched-chain aminoacids, have significantly different metabolites only in PH100; 2-methylpropan-1-ol (2) and 4-methylpentan-2-ol (9) appear in greater abundance in this condition. Several metabolites, such as methyl thiolacetate (255), 3-(methylthio)propanal (259), and S-methyl 3-methylbutanethioate (260), arise from the methionine pathway and significant difference is observed in PL100. Acrylate esters octyl acrylate (94) and 2-ethylhexyl acrylate (95) result from acrylate with 1-alkanols. Most significantly, different metabolites are observed in PH100. Metabolites from the metabolism of phenylalanine are also observed such as benzenemethanol (43), benzeneethanol (45), benzeneacetaldehyde (69), methyl benzoate (113), 1-phenyl acetate (116), 2-phenyl acetate (117), and 1-phenylbutan-1-one (222). Most significant differences are observed in PL100. However, significant differences are also noted in PH100 for benzenemethanol (43), benzeneethanol (45), 1-phenylethyl acetate (116), and 2-phenylethyl acetate (117). Aromatic metabolites 2-methylbenzaldehyde (70), 4-methylbenzaldehyde (71), 1,3-dimethylbenzene (160), naphthalene (169), biphenyl (173), and cyclohexanone (227) appear as statistically significant (*p* < 0.05) in PL100 and PH100. A large number of terpenoids have been identified resulting from the terpenoid skeleton biosynthesis pathway, probably through the non-mevalonate pathway. High metabolic activity is detected in PL100 and PH100 conditions to produce these metabolites. Metabolites 3-methylbut-3-en-1-ol (7) and 3-methylbut-2-en-1-ol (11) result from the precursors isopentenyl-PP and dimethylallyl-PP and are statistically significant (*p* < 0.05) in PL100 and PC25, respectively.

## Discussion

To the best of our knowledge, this is the first study reporting the volatile exometabolome of members of *Pedobacter*, a genus that has gained relevance in its application in various sectors, including in human health [9]. The volatile exometabolome of two phylogenetically close species, NL19 and MTCC6384, has been investigated focusing on the response to growth media composition, which was shown to influence antimicrobials production in NL19 [9]. The conditions tested (PL100, PH100, PL25, and PH25) show a plethora of metabolites generating complex data, as shown in Fig. 1 and above discussed. This figure reveals that, at first glance, all the conditions have a curiously similar profile. This was expected since NL19 and MTCC6384 belong to the same genus and are phylogenetically close [12]. A comparison of bubble plots shown in Fig. 1 reveals that peaks with the highest abundance were detected in PL100. However, bubble plots reflect a headspace snapshot of each condition tested, thus allowing a quick visualization of all MVOCs produced. Due to the large number of compounds identified, this analysis is difficult to be performed accurately, mainly because the information available in the literature is still scarce which, in turn, reveals the relevance of its study. A high percentage of alcohols, esters, hydrocarbons, and ketones was detected, which was also observed in other volatile exometabolome studies with bacteria [7, 16, 17]. Ketones and alcohols are more common than hydrocarbons and acids [18]. The absence of acids is highlighted, which may be due to the lack of glucose in the culture media. This was also reported by other authors, and in other bacterial species [19]. In the present study, the growth medium does not appear to affect the growth of MTCC6384 but it significantly affects the growth of NL19, as shown by the bar plot (Fig. 2d) and as previously reported [9]. This situation is also noted by the dendrogram that shows the PL100 clade isolated from the clade containing the other three conditions, indicating a high dissimilarity between PL100 and PL25 (Fig. 2a). PH100 and PH25 conditions have close relation in terms of similarity; however, PL25 and PH25 are even more closely similar (Fig. 2a). Especially for NL19, it can be suggested that slight modifications of the growth medium alter the metabolic profile. In particular, the lack of nitrogen seems to affect the primary metabolism, by decreasing the growth rate [20]. Although phylogenetically close, the growth rate of NL19 and MTCC6384 in the same culture medium is different, particularly in TSB100 [12].

Only 123 compounds were present in statistically significant (*p* < 0.05) amounts when compared to the respective controls. Figure 3 suggests that differences are consistent with species-dependent observations. This was expected and suggests that, despite the phylogenetic relationship and media composition, the metabolic profile is similar within the same strain. Two clusters reflect the high abundance of metabolites present in PH100 and PH25 when compared to PL100 and PH25. Figure 5 shows putative metabolic pathways probably involved in the formation of 80 metabolites. Despite the possible involvement of other possible pathways, these metabolites were associated with the following metabolic pathways: (i) branched-chain aminoacids, (ii) fatty acid metabolism, (iii) phenylalanine, (iv) degradation of aromatic compounds, (v) methionine metabolism, and (vi) non-mevalonate. However, a low number of metabolites can be associated with the respective metabolic pathway, since there is still little information available in the literature. A large number of volatile metabolites are produced from the primary metabolism, through carbohydrates and other substrates. These metabolites are simple and include acetone, acetate, butanone, butyrate, ethanol, among others, constituting the essential building blocks of other molecules. Aliphatic aminoacids are important (alanine, valine, leucine, and isoleucine), especially for fatty acid pathway and formation of branched metabolites. Those aminoacids form keto acids that are decarboxylated to furnish aldehydes with a similar side chain of the aminoacids [21]. The oxidation of the aldehydes can form 2-methylbutanoic acid, 3-methylbutanoic acid, and isobutyric acid, whereas the reduction can form alcohols such as 2-methylpropan-1-ol (2) and 4-methylpentan-2-ol (9) [22]. Both acids and alcohols can be linked by acyltransferases to form esters such as isoamyl acetate (81) and 2-methylbutyl acetate (82); these two esters were synthesized by linking leucine- and isoleucine-derived alcohols with acetate from primary metabolism, and have the same side chains as the abovementioned aminoacids. However, other known pathways lead to the formation of esters [23]. Further, aminoacids’ nitrogen can be used to form primary amines that are intermediates for several N-compounds [21].

As mentioned above, aliphatic aminoacids are associated with fatty acid pathways. Fatty acid metabolism generates great diversity of mVOCs with chains of different sizes and structural diversity. They start from acetyl-CoA and suffer several elongation cycles with malonyl-CoA until the desired length of the fatty acid is reached with an even number of carbons [21]. The same intermediates are involved in the reverse reaction through β-oxidation that leads to the formation of acetyl-CoA that is pumped into the citric acid cycle. The diversity of these metabolites is generated in two ways: (i) modifications of the starter unit, where the acetyl-CoA is replaced by intermediates derived from aliphatic aminoacids, resulting in branched metabolites [18]. These branched metabolites are frequently used in bacterial taxonomy [24]. Furthermore, modifications in the elongation step can occur such as decarboxylation steps in each extension intermediate and incorporation of methylmalonyl-CoA or ethylmalonyl-CoA instead of malonyl-CoA [18, 21, 25]. Decarboxylation during elongation may lead to the formation of alkanes such as nonane (137) and heptadecane (150), 1-alkenes, and methyl ketones such as pentan-2-one (1849, 4-methylpentan-2-one (187), hexan-2-one (189), 4-methylpent-3-en-2-one (190), 5-methylheptan-2-one (199), nonan-3-one (205), 6-methylhepta-3,5-diene-2-one (207), dodecan-2-one (215), and tridecan-2-one (217)) [21]. Several methyl ketones were formed by one of the starter units, further elongation, and decarboxylation [18]. Methyl ketones are highly diverse, and were already studied in the phylum Bacteroidetes [26]. The reduction of methyl ketones may form alcohols or esters such as 3-methylheptyl acetate (90). Incorporation of malonyl-CoA intercalated with methylmalonyl-CoA and further decarboxylation of this unit, during elongation, lead to the formation, by reduction, of ethyl ketones such as oct-1-en-3-ol (24), 5-methylhexan-3-one (191), 7-methyloctan-3-one (204), decan-3-one (210), and tridecan-3-one (216) and related alcohols, such as alcohol octan-3-ol (26). Acyl-CoA precursors can be hydrolysed and reduced or directly reduced to form acids, aldehydes such as pentanal (55), 2-ethylhexanal (59), and octanal (60), and 1-alkanols such as butan-1-ol (3), 2-methylpentan-1-ol (12), hexan-1-ol (14), 2-ethylhexan-1-ol (28), nonan-1-ol (35), decan-1-ol (40), and ethyl decanoate (103) during fatty acid metabolism [21]. The low percentage of unbranched aldehydes may be due to their high reactivity, being readily reduced to 1-alcohols [18]. Octanal (60) and nonane (137) can also result from oxidative stress [7]. In addition, nonane was the only metabolite identified in high abundance in PL100 condition. However, it is not possible to state that nonane results indeed from lipid peroxidation or fatty acid metabolism. The alcohol 5-methylheptan-1-ol (31) may result from the α-oxidation of the respective fatty acid [21]. Two metabolites, octyl acrylate (94) and 2-ethylhexyl acrylate (95), may arise from acrylate conjugation with respective 1-alkanol that can result in an acrylate ester [27]. Propionyl-CoA can be produced by several pathways, being the putative starting point for odd-numbered fatty acids or involved in the formation of citric acid cycle intermediates such as succinyl-CoA [28]. The high number of metabolites involved in this pathway suggests a high metabolic activity in the formation and degradation of fatty acids in all conditions tested. However, more significantly different metabolites are observed in PL100, PH100, and PH25 suggesting that this pathway is being used especially for obtaining energy. It seems also logical to think that the low lipid content present in TSB100 and PC25 media may trigger the activation of the biosynthetic machinery or enhance de novo biosynthesis of fatty acids, therefore mobilizing metabolites involved in fatty acid metabolism [29].

Metabolites resulting from phenylalanine are originated through the shikimate pathway. The metabolite benzeneethanol (45) is largely produced among bacteria and results from reduction of benzeneacetaldehyde (69) [21]. The conjugation of benzoyl-CoA with S-adenosyl methionine may originate methyl benzoate (113). The decarboxylation of phenylglyoxylate may originate benzenemethanol (43). The metabolite 1-phenylethyl acetate (116) can be hypothetically formed by conjugation of 1-phenylethanol with acetate [18]. The conjugation of 2-phenylehtanol may furnish a wide variety of aromatic esters and phenones (1-phenylbutan-1-one (222)) that are synthesized through benzoyl-CoA conjugation with fatty acids [21]. Metabolites and intermediates of phenylalanine are not only responsible for the synthesis of these volatiles but are also useful to replenish essential citric acid cycle intermediates. Other aromatic compounds resulting metabolites were also identified such as 2-methylbenzaldehyde (70), 4-methylbenzaldehyde (71), 1,3-dimethylbenzene (160), naphthalene (169), biphenyl (173), and cyclohexanone (227) [30]. In PL100 and PH100 media and for this pathway, a high amount of significantly different metabolites was detected.

S-compounds are common microbial volatiles and are mainly formed by sulphur aminoacid degradation, such as methionine. Transamination of methionine and dimethiolation of the intermediate α-keto-methylthiobutyric acid (KMBA) metabolite results in 2-oxobutanoate and methanethiol, the latter used for the formation of methyl thioesters such as methyl thiolacetate (255) and S-methyl 3-methylbutanethioate (260). 3-(Methylthio)propanal (259) arises from KMBA [18].

Monoterpenes are the most diverse group of microbial volatile compounds and are synthesized by two possible pathways. In the case of NL19 and MTCC6384, this synthesis seems to occur via a non-mevalonate pathway, originating sesquiterpenoids through farnesyl-PP and monoterpenoids through geranyl-PP. Metabolites 3-methylbut-3-en-1-ol (7) and 3-methylbut-2-en-1-ol (11) result from the primary precursors of the terpene backbone and are indicators of high production of terpenoids [18]. However, to date, few terpenes have been characterized in bacteria, so it is difficult to conclude whether many of them are produced by NL19 or MTCC6384.

Several metabolites were identified whose abundance is different between media (PL100-PL25 and PH100-PH25) (Table 1). Those metabolites that appear in statistically different abundances (*p* < 0.05) are highly abundant in PL25 and PH25. In NL19, several metabolites (2-methylbutyl acetate (82), but-1-en-1-yl-4-methylpentanoate (96), heptadecane (150), 4-phenylcyclohexene (172), and 1,1′-(1,3-propanediyl)bis-benzene (176)) are highly abundant in PL25 condition. Nonane (137), 4-methylpentan-2-one (187), levomenthol (286), and linalyl acetate (302) are increasingly abundant in PL100. 2-Methyldodecan-2-ol (37) and 2-tertiobutylthiophene (264) are less abundant in PL100 and PL25. Furthermore, in the case of MTCC6384, an increase in metabolic activity results in a decreased abundance of several metabolites such as 2-methylpentan-1-ol (12), 2-methyldodecan-2-ol (37), 2,4-Di-*tert-*butylphenol (47), octyl acrylate (949, 2-*tert-*butylcyclohexyl acetate (97), 1,3-dimethylbenzene (160), 1-ethyl-2,3-dimethylbenzene (168), and 4-methylhept-3-en-2-one (198) when compared to control. In general, metabolites that appear increased versus control condition occur in PH25 (4-phenylcyclohexene (172), 1,1′-(1-methyl-1,3-propanediyl)bis-benzene (176), and tridecan-3-one (216)) except metabolites pentanal (55) and carveol (294) whose abundance is high in PH100. Furthermore, pentanal (55) appears statistically abundant (*p* < 0.05) in PH100 and, at the same time, statistically less abundant (*p* < 0.05) in PH25. 3-Hydroxy-2,4,4-trimethylpentyl-2-methylpropanoate (101) and benzyl butyrate (119) appear in statistically high abundance (*p* < 0.05) in PH100, probably because there is a significant decrease in PH25. Other metabolites such as 4-methylpent-3-en-2-one (190) and 2-tertiobutylthiophene (264) decrease only in PH25, and both PH25 and PH100, respectively, but maintain a high abundance in PH25. However, there is an increased metabolic activity observed in PH100 that leads to a decrease in the abundance of the metabolites.

Of the smaller group of 24 metabolites (Fig. 4), only 2-methylpentan-1-ol (12), pentanal (55), 2-methylbutyl acetate (82), octyl acrylate (94), nonane (137), heptadecane (150), 1,3-dimethylbenzene (160), 4-methylpentan-2-one (187), 4-methylpent-3-en-2-one (190), tridecan-3-one (216), levomenthol (286), carveol (294), and linalyl acetate (302) could be associated with metabolic pathways (Fig. 5). Accordingly, for NL19, the abundance of 2-methylbutyl acetate (82) and heptadecane (150) increased in PL25 indicating increased metabolic activity of branched-chain aminoacids and fatty acid metabolic pathway, respectively. The abundance of 4-methylpentan-2-one (187) and nonane (137), which results from fatty acid metabolism increased in PL100. Levomenthol (286) and linalyl acetate (302) result from terpenoid biosynthesis and this pathway is particularly active in PL100, probably due to the high availability of resources. For MTCC6384 strain, the abundance of 2-methylpentan-1-ol (12), octyl acrylate (94), and 1,3-dimethylbenzene (160) decreased in PH100, whereas the abundance of tridecan-3-one (216) increased in PH25. These compounds are mainly involved in fatty acid pathway, and suggest an increase of metabolic activity to replenish cellular energy, especially in the PH100. Metabolite 1,3-dimethylbenzene (160) is related to the degradation of aromatic metabolites whose pathway leads to the formation of citric acid cycle intermediates. Pentanal (55), which is also related to the metabolism of fatty acids, is highly abundant in PH100. Carveol (294) results from terpenoid biosynthesis, suggesting an increase in the activity of this pathway in PH100. Metabolite 4-methylpent-3-en-2-one (190) decreases in PH25 but remains in statistically significant abundance (*p* < 0.05) relative to PH100, and it is also related to the fatty acid metabolism pathway. This mixed behaviour does not allow conclusions to be drawn about the behaviour of MTC6384 in PH100 and PH25. However, due to the decreased abundance of many metabolites in PH100, it can be suggested that there is a high metabolic activity in this condition, in comparison to PH25, leading to the mobilization of metabolites involved in fatty acid metabolism, probably to replenish cellular energy. For NL19, metabolites with putative pathways are not sufficient to explain the reason for such different behaviour between PL100 and PL25. However, a slightly increased metabolic activity is observed in PL25 and the possible metabolic pathways identified, despite being few, suggest that there is an increase in the mobilization of metabolites probably involved in important metabolic functions such as the citric acid cycle and others, due to starvation.

Overall, the results presented here suggest that a decrease in the casein peptone concentration to 25% appears to have a strong impact on microbial growth and in metabolic profiles of NL19 and MTCC6384. Apparently, the reduction of this nitrogen source leads, respectively, to an increase and decrease in the metabolic activity of cells growing in PL25 and PH25. This metabolic alteration translates into an increase or decrease of metabolites involved in the citric acid cycle, which may be involved in cellular energy production processes and other processes important for the maintenance of normal cellular functions.

## Concluding remarks

To the best of our knowledge, this is the first study conducted on bacteria of the genus *Pedobacter*, contributing to a better understanding of how the composition of the culture medium impacts the metabolic profile and, therefore, the volatile exometabolome of two closely related species, NL19 and MTCC6384. Analysis of the volatile exometabolome revealed that the decrease in nitrogen source concentration had a strong impact on energy-producing metabolism. Clearly, NL19 is most affected by this effect, while MTCC6384 adapts best to this change. A detailed analysis of 123 metabolites (compounds with at least on condition that was statistically different (*p* < 0.05) to the respective control media) showed that the metabolic activity of the cells is markedly increased in PL25. This is explained by the activation of accessory pathways responsible for energy production, probably due to the stress caused by starvation. In PH100, a slight decrease in cell biomass is observed, and as such, increased activity may be a result of excess nitrogen in growth media, which in turn may activate several different pathways involved in aminoacid metabolism. The fact that we identified many metabolites that did not match in pathways databases and the literature made the study very difficult. Thus, even more efforts are needed to improve and contribute to feeding these databases, which will certainly facilitate access to information in the literature. With this study, it was possible to understand the impact of the media on the growth and metabolism of microorganisms. Slight changes in the culture medium can lead to alterations in the primary metabolism which in turn can alter the secondary metabolism, leading to the formation of biotechnologically relevant metabolites. Finally, it is important to point out that the implemented methodology based on HS-SPME/GC × GC-ToFMS was shown to be suitable for the profiling of exometabolome from bacteria of the genus *Pedobacter*, which represent the crucial step in the construction of a metabolomics workflow. We are just at the tip of the iceberg, and further and more comprehensive studies are needed. Still, the information gathered allows the creation of an omics pipeline that can certainly be useful for other studies and approaches such as genomics, transcriptomics, and proteomics.

## Supplementary Information

Below is the link to the electronic supplementary material.

Supplementary file1 (DOCX 1087 KB)

Supplementary file2 (XLSX 141 KB)
