# Supplementary material for: Linking Pedobacter lusitanus NL19 volatile exometabolome with growth medium composition: what can we learn using comprehensive two-dimensional gas chromatography coupled to time-of-flight mass spectrometry?
Source: Anal Bioanal Chem. 2023 Jan 11;415(13):2613–27. doi: 10.1007/s00216-022-04505-6 (PMC10149447; doi:10.1007/s00216-022-04505-6)
Supplement: Supplementary file 1 — Supplementary file1 (DOCX 1087 KB) [file 216_2022_4505_MOESM1_ESM.docx]

**Linking *Pedobacter lusitanus* NL19 volatile exometabolome with growth medium composition: what can we learn using comprehensive two-dimensional gas chromatography coupled to time-of-flight mass spectrometry?**

**Gonçalo Figueiredo^a^, Carina Pedrosa Costa^b^, Joana Lourenço^a^, Tânia Caetano^a^, Sílvia M. Rocha^b*^, Sónia Mendo^a*^**

^a^ CESAM and Department of Biology, University of Aveiro, Campus Universitario de Santiago, 3810-193, Aveiro, Portugal.

^b^ LAQV-REQUIMTE & Department of Chemistry, University of Aveiro, Campus Universitário Santiago,3810-193 Aveiro, Portugal

^*^ Corresponding authors emails:

Sónia Mendo; [smendo@ua.pt](mailto:smendo@ua.pt)

Sílvia M. Rocha; [smrocha@ua.pt](mailto:smrocha@ua.pt)

**Supplementary Information**

Figure S1


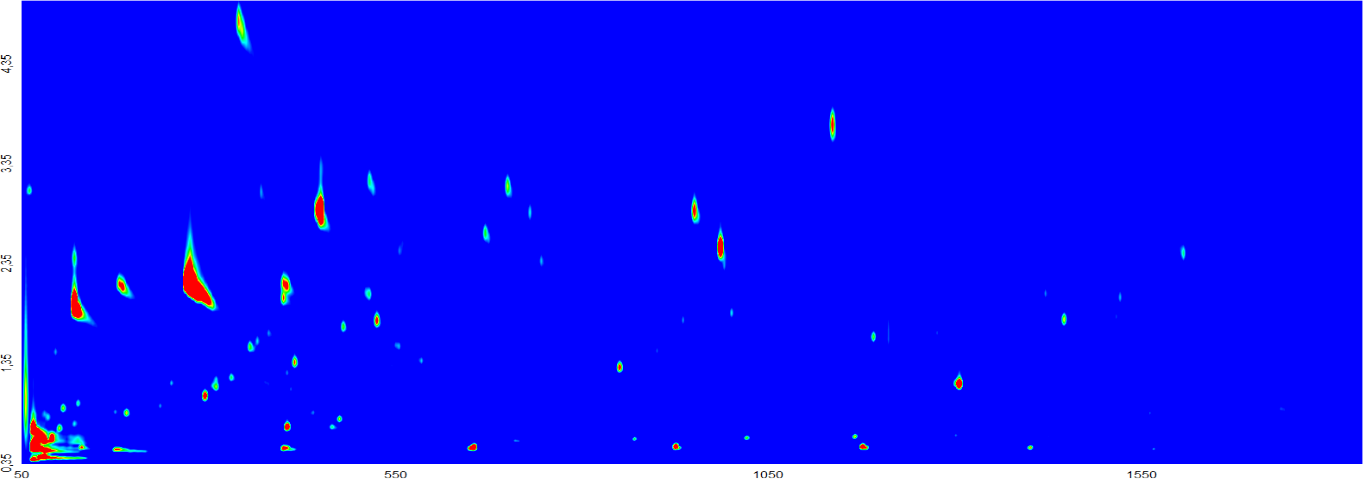


**PL100**


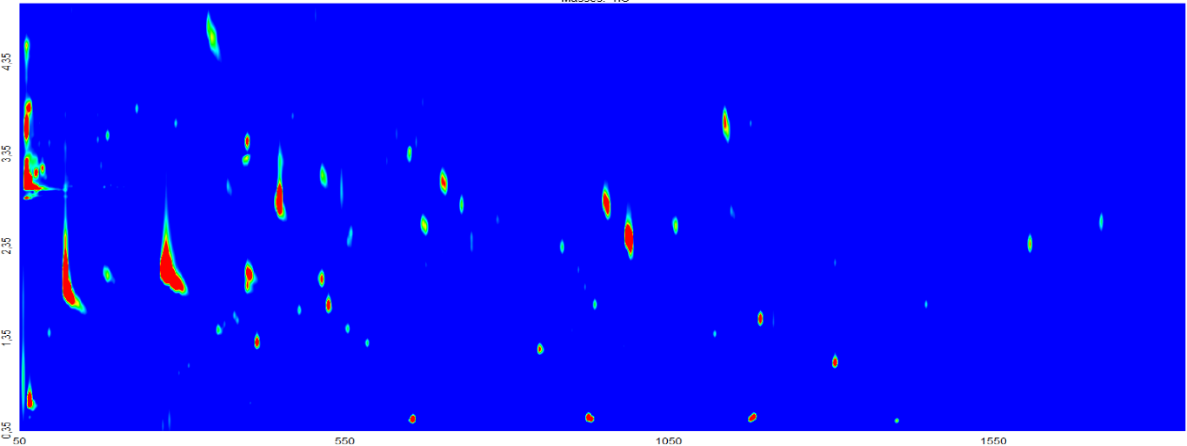


**PH100**


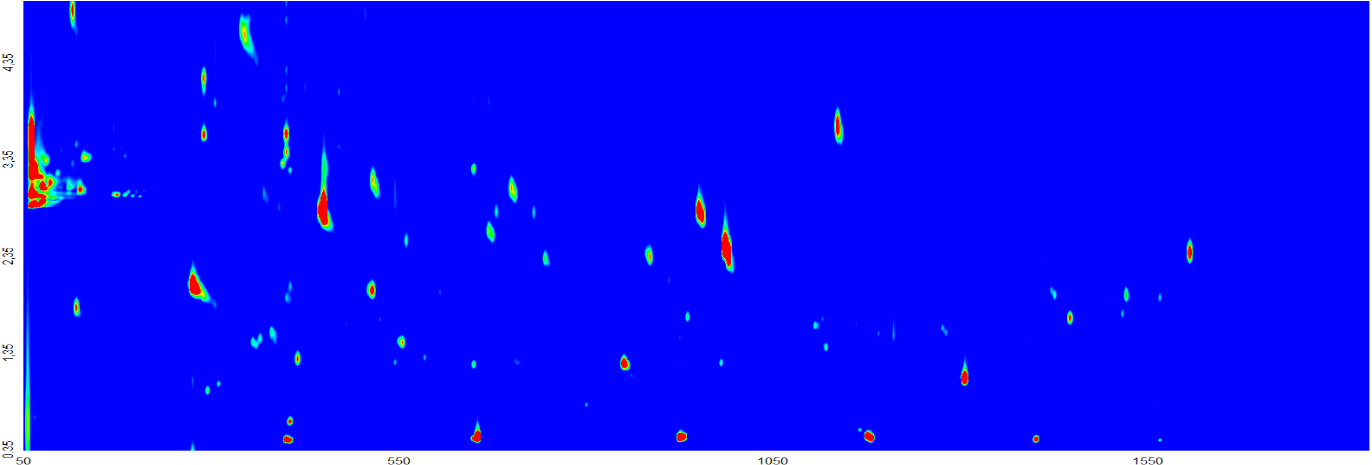


**PL25**


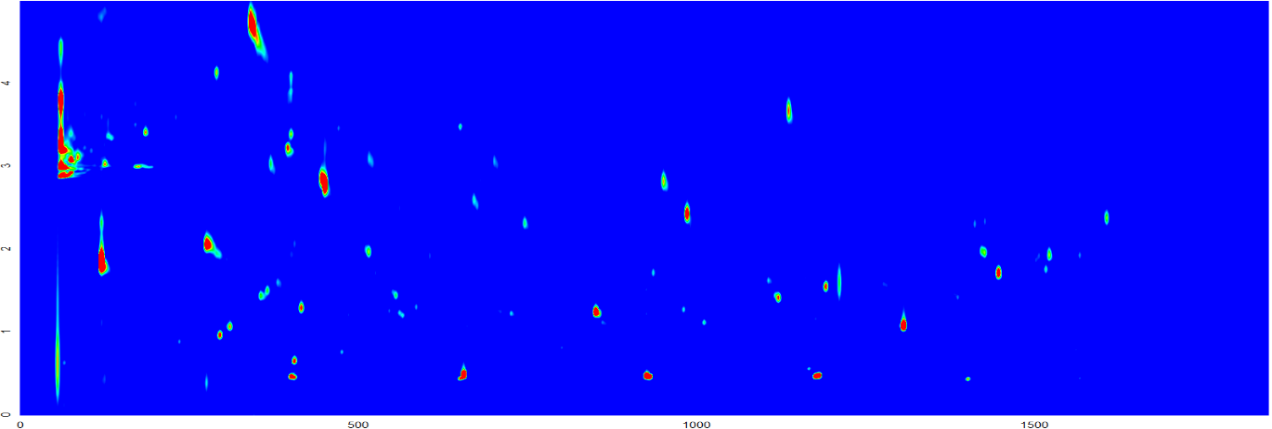


**PH25**

Figure S1 GC×GC-ToFMS total ion chromatogram contour plots of the headspace volatile components extracted from the three assessed conditions: *P. lusitanus* TSB100% (PL100), *P. lusitanus* PC25% (PL25), *P himalayensis* TSB100% (PH100) and *P. himalayensis* PC25% (PH25). The X axis is the 1^st^ Dimension, in seconds, that is translated by the decrease of volatility over time. The Y axis is the 2^nd^ dimension, in seconds, that is translated by the increase of polarity over time of the analytes.

Table S1

| **Table S1** Full data set of the 320 putatively identified metabolites from the headspace of all samples under study. Statistically different compounds to at least one of the respective controls are marked as bold. | | | | | | | | | | | | | | | | | |
| --- | --- | --- | --- | --- | --- | --- | --- | --- | --- | --- | --- | --- | --- | --- | --- | --- | --- |
| **ID** | **^1^*t*_R_ ^a^ (s)** | **^2^*t*_R_ ^a^ (s)** | | **Compound** | | | | **CAS number** | | | **Formula** | **RI_Calc_ ^b^** | | **RI_Lit_ ^c^** | | | |
| ***Alcohols* (15.9%)** | | | | | | | | | | | | | | | | | |
| *Aliphatic* | | | | | | | | | | | | | | | | | |
| 1 | 80 | 0.870 | | Butan-2-ol | | | | 78-92-2 | | | C4H10O | 626 | | 601 | | | [1] |
| **2** | **85** | **1.160** | | **2-Methylpropan-1-ol** | | | | **78-83-1** | | | **C4H10O** | **633** | | **612** | | | [1] |
| **3** | **95** | **1.480** | | **Butan-1-ol** | | | | **71-36-3** | | | **C4H10O** | **662** | | **668** | | | [2] |
| 4 | 105 | 1.190 | | Pentan-3-ol | | | | 584-02-1 | | | C5H12O | 685 | | 675 | | | [1] |
| 5 | 105 | 1.270 | | Pentan-2-ol | | | | 6032-29-7 | | | C5H12O | 685 | | 675 | | | [1] |
| 6 | 115 | 1.120 | | 2-Methylpentan-2-ol | | | | 590-36-3 | | | C6H14O | 709 | | 704 | | | [3] |
| **7** | **120** | **2.430** | | **3-Methylbut-3-en-1-ol** | | | | **763-32-6** | | | **C5H10O** | **723** | | **708** | | | [1] |
| 8 | 120 | 1.970 | | 3-Methylbutan-1-ol | | | | 123-51-3 | | | C5H12O | 722 | | 718 | | | [1] |
| **9** | **135** | **1.460** | | **4-Methylpentan-2-ol** | | | | **108-11-2** | | | **C6H14O** | **756** | | **749** | | | [1] |
| 10 | 140 | 2.280 | | Pentan-1-ol | | | | 71-41-0 | | | C5H12O | 770 | | 760 | | | [1] |
| **11** | **150** | **3.420** | | **3-Methylbut-2-en-1-ol** | | | | **556-82-1** | | | **C5H10O** | **796** | | **782** | | | [1] |
| **12** | **195** | **2.500** | | **2-Methylpentan-1-ol** | | | | **105-30-6** | | | **C6H14O** | **843** | | **843** | | | [4] |
| 13 | 210 | 1.440 | | 3-Ethylpentan-3-ol | | | | 597-49-9 | | | C7H16O | 856 | | 851 | | | [5] |
| **14** | **235** | **2.950** | | **Hexan-1-ol** | | | | **111-27-3** | | | **C6H14O** | **883** | | **878** | | | [1] |
| 15 | 260 | 1.940 | | Heptan-3-ol | | | | 589-82-2 | | | C7H16O | 905 | | 901 | | | [1] |
| 16 | 280 | 1.880 | | 2,5-Dimethylhexane-2,5-diol | | | | 110-03-2 | | | C8H18O2 | 920 | | 916 | | | [6] |
| 17 | 290 | 1.870 | | 3-Ethylhex-5-en-3-ol | | | | 1907-46-6 | | | C8H16O | 927 | | - | | | - |
| 18 | 300 | 1.690 | | 2-Methylheptan-2-ol | | | | 625-25-2 | | | C8H18O | 935 | | 916 | | | [7] |
| 19 | 325 | 3.180 | | 4-Methylhexan-1-ol | | | | 818-49-5 | | | C7H16O | 954 | | 950 | | | [8] |
| 20 | 335 | 2.140 | | 4-Methylheptan-2-ol | | | | 56298-90-9 | | | C8H18O | 961 | | - | | | - |
| 21 | 360 | 2.280 | | Octan-2-ol | | | | 123-96-6 | | | C8H18O | 979 | | 964 | | | [1] |
| 22 | 365 | 3.220 | | Heptan-1-ol | | | | 111-70-6 | | | C7H16O | 984 | | 975 | | | [1] |
| **23** | **365** | **1.580** | | **8-Methylnonane-1,8-diol** | | | | **54725-73-4** | | | **C10H22O2** | **983** | | **-** | | | **-** |
| **24** | **370** | **3.080** | | **Oct-1-en-3-ol** | | | | **3391-86-4** | | | **C8H16O** | **987** | | **980** | | | [1] |
| 25 | 390 | 2.930 | | 6-Methylheptan-1-ol | | | | 1653-40-3 | | | C8H18O | 1002 | | 996 | | | [1] |
| **26** | **395** | **2.080** | | **Octan-3-ol** | | | | **589-98-0** | | | **C8H18O** | **1004** | | **996** | | | [1] |
| **27** | **435** | **3.270** | | **3-Ethyl-4-methylpentan-1-ol** | | | | **100431-87-6** | | | **C8H18O** | **1030** | | **1023** | | | [1] |
| **28** | **445** | **2.890** | | **2-Ethylhexan-1-ol** | | | | **104-76-7** | | | **C8H18O** | **1036** | | **1029** | | | [1] |
| 29 | 455 | 1.560 | | 3-Ethylheptan-3-ol | | | | 19780-41-7 | | | C9H20O | 1042 | | - | | | - |
| **30** | **475** | **0.810** | | **2,5-Dimethylhexane-3,4-diol** | | | | **22607-11-0** | | | **C8H18O2** | **1054** | | **-** | | | **-** |
| **31** | **475** | **3.140** | | **5-Methylheptan-1-ol** | | | | **7212-53-5** | | | **C8H18O** | **1055** | | **1051** | | | [1] |
| 32 | 515 | 3.200 | | Octan-1-ol | | | | 111-87-5 | | | C8H18O | 1080 | | 1079 | | | [1] |
| 33 | 565 | 2.210 | | 4-Methylhexan-2-ol | | | | 2313-61-3 | | | C7H16O | 1110 | | - | | | - |
| **34** | **665** | **1.370** | | **3,4-Diethylhexan-3-ol** | | | | **19398-78-8** | | | **C10H22O** | **1171** | | **-** | | | **-** |
| **35** | **680** | **2.900** | | **Nonan-1-ol** | | | | **143-08-8** | | | **C9H20O** | **1181** | | **1173** | | | [1] |
| 36 | 725 | 2.070 | | Decan-2-ol | | | | 1120-06-5 | | | C10H22O | 1208 | | 1209 | | | [9] |
| **37** | **735** | **1.370** | | **2-Methyldodecan-2-ol** | | | | **1653-37-8** | | | **C13H28O** | **1213** | | **-** | | | **-** |
| 38 | 745 | 2.410 | | 2-Propylheptan-1-ol | | | | 10042-59-8 | | | C10H22O | 1220 | | 1219 | | | [1] |
| **39** | **765** | **1.440** | | **2-Methyldecan-2-ol** | | | | **3396-02-9** | | | **C11H24O** | **1232** | | **1231** | | | [10] |
| **40** | **840** | **2.640** | | **Decan-1-ol** | | | | **112-30-1** | | | **C10H22O** | **1280** | | **1278** | | | [1] |
| 41 | 885 | 1.920 | | Undecan-2-ol | | | | 1653-30-1 | | | C11H24O | 1308 | | 1301 | | | [1] |
| 42 | 1035 | 1.810 | | Dodecan-2-ol | | | | 10203-28-8 | | | C12H26O | 1412 | | 1414 | | | [1] |
|  |  |  | |  | | | |  | | |  |  | |  | | |  |
| *Aromatic* | | | | | | | | | | | | | | | | | |
| **43** | **490** | **4.540** | | **Benzenemethanol** | | | | **100-51-6** | | | **C7H8O** | **1065** | | **1049** | | | [1] |
| **44** | **545** | **3.050** | | **α,α-Dimethylbenzenemethanol** | | | | **617-94-7** | | | **C9H12O** | **1099** | | **1091** | | | [1] |
| **45** | **600** | **3.490** | | **Benzeneethanol** | | | | **60-12-8** | | | **C8H10O** | **1132** | | **1120** | | | [1] |
| **46** | **1190** | **1.630** | | **Butylated hydroxytoluene** | | | | **128-37-0** | | | **C15H24O** | **1523** | | **1519** | | | [1] |
| **47** | **1210** | **1.670** | | **2,4-Di-*tert*-butylphenol** | | | | **96-76-4** | | | **C14H22O** | **1537** | | **1525** | | | [11] |
|  |  |  | |  | | | |  | | |  |  | |  | | |  |
| *Cyclic* | | | | | | | | | | | | | | | | | |
| 48 | 295 | 1.570 | | 1-Cyclopentylpropan-1-ol | | | | 19833-89-7 | | | C8H16O | 931 | | - | | | - |
| **49** | **650** | **2.270** | | **α,α-4-Trimethylcyclohexanemethanol** | | | | **498-81-7** | | | **C10H20O** | **1162** | | **1161** | | | [12] |
| 50 | 745 | 1.440 | | α,α-Dimethyl-2-methylenecyclopropanemethanol | | | | 126434-25-1 | | | C7H12O | 1220 | | - | | | - |
| 51 | 1500 | 1.960 | | Tricyclo[7.4.1.1(2,7)]pentadeca-2,4,6,9,11,13-hexaene-8-ol | | | | 104739-73-3 | | | C15H140 | 1773 | | - | | | - |
|  | | |  | |  |  |  | |  |  | | |  | |  |  |  |
| ***Aldehydes* (7.5%)** | | | | | | | | | | | | | | | | | |
| *Aliphatic* | | | | | | | | | | | | | | | | | |
| 52 | 60 | 0.420 | | Acetaldehyde | | | | 75-07-0 | | | C2H4O | 577 | | 548 | | | [1] |
| 53 | 85 | 3.210 | | 3-Methylbutanal | | | | 590-86-3 | | | C5H10O | 643 | | 633 | | | [1] |
| 54 | 95 | 0.920 | | But-2-enal | | | | 4170-30-3 | | | C4H6O | 661 | | 657 | | | [13] |
| **55** | **110** | **3.090** | | **Pentanal** | | | | **110-62-3** | | | **C5H10O** | **701** | | **699** | | | [14] |
| 56 | 125 | 1.070 | | 2-Methyl-2-butenal | | | | 1115-11-3 | | | C5H8O | 732 | | 728 | | | [1] |
| 57 | 160 | 0.940 | | Hexanal | | | | 66-25-1 | | | C6H12O | 804 | | 801 | | | [1] |
| 58 | 260 | 1.150 | | Heptanal | | | | 111-71-7 | | | C7H14O | 905 | | 901 | | | [1] |
| **59** | **330** | **1.000** | | **2-Ethylhexanal** | | | | **123-05-7** | | | **C8H16O** | **956** | | **953** | | | [1] |
| **60** | **405** | **1.270** | | **Octanal** | | | | **124-13-0** | | | **C8H16O** | **1010** | | **1001** | | | [1] |
| 61 | 490 | 1.849 | | Oct-2-enal | | | | 2363-89-5 | | | C8H14O | 1064 | | 1062 | | | [1] |
| 62 | 565 | 1.290 | | Nonanal | | | | 124-19-6 | | | C9H18O | 1110 | | 1106 | | | [1] |
| 63 | 725 | 1.300 | | Decanal | | | | 112-31-2 | | | C10H20O | 1207 | | 1207 | | | [1] |
| 64 | 815 | 1.711 | | Dec-2-enal | | | | 3913-81-3 | | | C10H18O | 1264 | | 1260 | | | [1] |
| **65** | **905** | **2.569** | | **Deca-2,4-dienal** | | | | **2363-88-4** | | | **C10H16O** | **1323** | | **1295** | | | [1] |
| 66 | 975 | 1.630 | | Undec-2-enal | | | | 2463-77-6 | | | C11H20O | 1370 | | 1364 | | | [1] |
| 67 | 1040 | 1.260 | | Dodecanal | | | | 112-54-9 | | | C12H24O | 1415 | | 1412 | | | [15] |
|  |  |  | |  | | | |  | | |  |  | |  | | |  |
| *Aromatic* | | | | | | | | | | | | | | | | | |
| 68 | 340 | 4.780 | | Benzaldehyde | | | | 100-52-7 | | | C7H6O | 967 | | 965 | | | [1] |
| **69** | **475** | **0.400** | | **Benzeneacetaldehyde** | | | | **122-78-1** | | | **C8H8O** | **1053** | | **1049** | | | [15] |
| **70** | **505** | **4.300** | | **2-Methylbenzaldehyde** | | | | **529-20-4** | | | **C8H8O** | **1075** | | **1067** | | | [2] |
| **71** | **525** | **4.500** | | **4-Methylbenzaldehyde** | | | | **104-87-0** | | | **C6H8O** | **1087** | | **1085** | | | [1] |
| 72 | 660 | 0.730 | | 2-Phenylpropenal | | | | 4432-63-7 | | | C9H8O | 1167 | | 1163 | | | [1] |
| **73** | **740** | **4.400** | | **3,5-Dimethylbenzaldehyde** | | | | **5779-95-3** | | | **C9H10O** | **1218** | | **1219** | | | [1] |
| 74 | 885 | 3.080 | | 4-(*t*-Butyl)benzaldehyde | | | | 939-97-9 | | | C11H14O | 1309 | | 1308 | | | [1] |
| **75** | **1500** | **3.250** | | **3,5-di-*tert*-Butyl-4-hydroxybenzaldehyde** | | | | **1620-98-0** | | | **C15H22O2** | **1775** | | **1774** | | | [16] |
| ***Esters* (15.6%)** | | | | | | | | | | | | | | | | | |
| *Aliphatic* | | | | | | | | | | | | | | | | | |
| 76 | 80 | 0.570 | | Ethyl acetate | | | | 141-78-6 | | | C4H8O2 | 625 | | 612 | | | [17] |
| **77** | **105** | **3.330** | | **Methyl methacrylate** | | | | **80-62-6** | | | **C5H8O2** | **690** | | **685** | | | [1] |
| 78 | 175 | 0.880 | | Butyl acetate | | | | 123-86-4 | | | C6H12O2 | 821 | | 818 | | | [1] |
| 79 | 205 | 3.440 | | Ethyl isovalerate | | | | 108-64-5 | | | C7H14O2 | 853 | | 856 | | | [18] |
| 80 | 205 | 0.819 | | 2-Pentyl acetate | | | | 626-38-0 | | | C7H14O2 | 851 | | 854 | | | [1] |
| **81** | **230** | **3.850** | | **Isoamyl acetate** | | | | **123-92-2** | | | **C7H14O2** | **879** | | **877** | | | [1] |
| **82** | **235** | **4.140** | | **2-Methylbutyl acetate** | | | | **624-41-9** | | | **C7H14O2** | **884** | | **883** | | | [1] |
| 83 | 335 | 0.880 | | Isobutyl butyrate | | | | 539-90-2 | | | C8H16O2 | 960 | | 958 | | | [1] |
| 84 | 355 | 1.018 | | 2-Methylpentyl acetate | | | | 7789-99-3 | | | C8H16O2 | 975 | | 949 | | | [1] |
| 85 | 395 | 0.970 | | Butyl butyrate | | | | 109-21-7 | | | C8H16O2 | 1004 | | 995 | | | [1] |
| 86 | 400 | 1.020 | | Ethyl hexanoate | | | | 123-66-0 | | | C8H16O2 | 1007 | | 1001 | | | [1] |
| 87 | 425 | 1.120 | | Hexyl acetate | | | | 142-92-7 | | | C8H16O2 | 1023 | | 1024 | | | [19] |
| 88 | 485 | 0.960 | | Isoamyl butyrate | | | | 106-27-4 | | | C9H18O2 | 1060 | | 1056 | | | [1] |
| 89 | 555 | 1.750 | | 2-Butoxyethyl acetate | | | | 112-07-2 | | | C8H16O3 | 1104 | | 1096 | | | [1] |
| **90** | **640** | **1.050** | | **3-Methylheptyl acetate** | | | | **72218-58-7** | | | **C10H20O2** | **1155** | | **1156** | | | [1] |
| **91** | **675** | **1.030** | | **Isononyl acetate** | | | | **40379-24-6** | | | **C11H22O2** | **1176** | | **-** | | | **-** |
| 92 | 700 | 2.290 | | Dimethyl 2,4-dimethyl-3-oxopentanedioate | | | | 2121-68-8 | | | C9H16O4 | 1192 | | 1213 | | | [1] |
| 93 | 715 | 1.060 | | Ethyl octanoate | | | | 106-32-1 | | | C10H20O2 | 1201 | | 1195 | | | [1] |
| **94** | **720** | **1.130** | | **Octyl acrylate** | | | | **2499-59-4** | | | **C11H20O2** | **1204** | | **-** | | | **-** |
| **95** | **770** | **1.110** | | **2-Ethylhexyl acrylate** | | | | **103-11-7** | | | **C11H20O2** | **1235** | | **1230** | | | [1] |
| **96** | **830** | **2.030** | | **But-1-en-1-yl 4-methylpentanoate** | | | | **-** | | | **C10H18O2** | **1273** | | **-** | | | **-** |
| **97** | **860** | **1.210** | | **2-*tert*-Butylcyclohexyl acetate** | | | | **88-41-5** | | | **C12H22O2** | **1291** | | **-** | | | **-** |
| 98 | 910 | 2.170 | | α,α-Dimethylbenzeneethanol acetate | | | | 151-05-3 | | | C13H22O | 1326 | | - | | | - |
| 99 | 925 | 1.300 | | 4-*tert*-Butylcyclohexyl acetate (isomer) | | | | 32210-23-4 | | | C12H16O2 | 1326 | | 1320 | | | [1] |
| 100 | 940 | 3.230 | | Methyl α-methylenebenzenepropanoate | | | | 3070-71-1 | | | C11H12O2 | 1347 | | 1339 | | | [1] |
| **101** | **985** | **2.570** | | **3-Hydroxy-2,4,4-trimethylpentyl-2-methylpropanoate** | | | | **74367-34-3** | | | **C12H24O3** | **1378** | | **1376** | | | [1] |
| 102 | 990 | 2.370 | | 2-(2-Butoxyethoxy)ethyl acetate | | | | 124-17-4 | | | C10H20O4 | 1381 | | 1366 | | | [20] |
| **103** | **1025** | **1.051** | | **Ethyl decanoate** | | | | **110-38-3** | | | **C12H24O2** | **1404** | | **1395** | | | [1] |
| 104 | 1115 | 1.770 | | Diisopropyl adipate | | | | 6938-94-9 | | | C12H22O4 | 1469 | | 1464 | | | [21] |
| 105 | 1300 | 3.260 | | Hexamethylene diacrylate | | | | 13048-33-4 | | | C12H18O4 | 1603 | | 1604 | | | [22] |
| 106 | 1300 | 1.160 | | 2-Methylpropanoic acid, 1-(1,1-dimethylethyl)-2-methylpropane-1,3-diyl ester | | | | 74381-40-1 | | | C16H30O4 | 1601 | | - | | | - |
| **107** | **1345** | **0.930** | | **Isopropyl laurate** | | | | **10233-13-3** | | | **C15H30O2** | **1637** | | **1630** | | | [1] |
| 108 | 1380 | 2.910 | | Methyl dihydrojasmonate | | | | 24851-98-7 | | | C13H22O3 | 1666 | | 1650 | | | [23] |
| 109 | 1415 | 0.810 | | Nonyl 2-methylpropanoate | | | | 10522-34-6 | | | C13H26O2 | 1693 | | - | | | - |
| 110 | 1505 | 1.690 | | Dibutyl adipate | | | | 105-99-7 | | | C14H26O4 | 1778 | | 1766 | | | [21] |
| 111 | 1560 | 0.870 | | Isopropyl myristate | | | | 110-27-0 | | | C17H34O2 | 1834 | | 1835 | | | [24] |
| 112 | 1730 | 0.860 | | Isopropyl palmitate | | | | 142-91-6 | | | C19H38O2 | 2025 | | 2024 | | | [10] |
|  |  |  | |  | | | |  | | |  |  | |  | | |  |
| *Aromatic* | | | | | | | | | | | | | | | | | |
| **113** | **550** | **3.660** | | **Methyl benzoate** | | | | **93-58-3** | | | **C8H8O2** | **1102** | | **1101** | | | [1] |
| 114 | 670 | 3.980 | | Benzyl acetate | | | | 140-11-4 | | | C9H10O2 | 1175 | | 1170 | | | [25] |
| 115 | 675 | 2.940 | | Ethyl benzoate | | | | 93-89-0 | | | C9H10O2 | 1178 | | 1173 | | | [1] |
| **116** | **715** | **3.030** | | **1-Phenylethyl acetate** | | | | **93-92-5** | | | **C10H12O2** | **1202** | | **1206** | | | [26] |
| **117** | **815** | **3.500** | | **2-Phenylethyl acetate** | | | | **103-45-7** | | | **C10H12O2** | **1265** | | **1260** | | | [1] |
| 118 | 920 | 2.250 | | Isobutyl benzoate | | | | 120-50-3 | | | C11H14O2 | 1332 | | 1326 | | | [1] |
| **119** | **950** | **2.800** | | **Benzyl butyrate** | | | | **103-37-7** | | | **C11H14O2** | **1354** | | **1345** | | | [27] |
| 120 | 955 | 1.640 | | Cynnamil propionate | | | | 103-56-0 | | | C12H14O2 | 1356 | | 1351 | | | [1] |
| 121 | 1025 | 2.310 | | 2-Methylbenzenemethanol acetate | | | | 17373-93-2 | | | C10H12O2 | 1405 | | 1395 | | | [1] |
| 122 | 1115 | 2.525 | | Ethyl α-ethylbenzeneacetate | | | | 119-43-7 | | | C12H16O2 | 1470 | | 1458 | | | [1] |
| **123** | **1160** | **1.730** | | **1,1-Dimethyl-2-phenylethyl butanoate** | | | | **10094-34-5** | | | **C14H20O2** | **1501** | | **1495** | | | [1] |
| 124 | 1440 | 1.690 | | 2-Octyl benzoate | | | | 6938-51-8 | | | C15H22O2 | 1716 | | - | | | - |
|  |  |  | |  | | | |  | | |  |  | |  | | |  |
| *Cyclic* | | | | | | | | | | | | | | | | | |
| 125 | 1130 | 4.290 | | γ-Decalactone | | | | 706-14-9 | | | C10H18O2 | 1482 | | 1463 | | | [15] |
|  |  |  | |  | | | |  | | |  |  | |  | | |  |
| ***Ethers* (3.4%)** | | | | | | | | | | | | | | | | | |
| *Aliphatic* | | | | | | | | | | | | | | | | | |
| **126** | **80** | **0.440** | | **2-Ethoxy-2-methylpropane** | | | | **637-92-3** | | | **C6H14O** | **625** | | **620** | | | [28] |
| **127** | **275** | **3.280** | | **2-Butoxyethanol** | | | | **111-76-2** | | | **C6H14O2** | **917** | | **910** | | | [29] |
| 128 | 315 | 2.040 | | 1-Butoxy-2-propanol | | | | 5131-66-8 | | | C7H16O2 | 946 | | 943 | | | [1] |
| 129 | 715 | 4.670 | | 2-(2-Butoxyethoxy)ethanol | | | | 112-34-5 | | | C8H18O3 | 1203 | | 1196 | | | [1] |
| 130 | 740 | 1.090 | | 3-(2,2-Dimethylpropoxy)-2-butanol | | | | 74793-66-1 | | | C9H20O2 | 1216 | | - | | | - |
| 131 | 1300 | 2.730 | | 1-*tert*-Butoxy-2-ethoxyethane | | | | 51422-54-9 | | | C8H18O2 | 1602 | | - | | | - |
| 132 | 1385 | 0.730 | | Dioctyl ether | | | | 629-82-3 | | | C16H34O | 1669 | | - | | | - |
|  |  |  | |  | | | |  | | |  |  | |  | | |  |
| *Aromatic* | | | | | | | | | | | | | | | | | |
| **133** | **505** | **1.450** | | **1,1-Dimethylethoxybenzene** | | | | **6669-13-2** | | | **C10H14O** | **1073** | | **1074** | | | [30] |
| 134 | 685 | 1.540 | | 1,1-Dimethylethoxymethylbenzene | | | | 3459-80-1 | | | C11H16O | 1183 | | 1184 | | | [1] |
| 135 | 790 | 1.620 | | Thymol methyl ether | | | | 1076-56-8 | | | C11H16O | 1248 | | 1235 | | | [31] |
| 136 | 1030 | 3.540 | | Diphenyl ether | | | | 101-84-8 | | | C12H10O | 1410 | | 1402 | | | [1] |
|  |  |  | |  | | | |  | | |  |  | |  | | |  |
| ***Hydrocarbons* (13.8%)** | | | | | | | | | | | | | | | | | |
| *Aliphatic* | | | | | | | | | | | | | | | | | |
| **137** | **255** | **0.510** | | **Nonane** | | | | **111-84-2** | | | **C9H20** | **898** | | **900** | | | [1] |
| 138 | 390 | 0.550 | | Decane | | | | 124-18-5 | | | C10H22 | 1000 | | 1000 | | | [1] |
| 139 | 550 | 0.570 | | Undecane | | | | 1120-21-4 | | | C12H26 | 1100 | | 1106 | | | [1] |
| **140** | **610** | **3.110** | | **3,3-Dimethyloct-1-ene** | | | | **74511-51-6** | | | **C10H20** | **1138** | | **1151** | | | [32] |
| **141** | **635** | **2.900** | | **6-Methyloct-1-ene** | | | | **13151-10-5** | | | **C9H18** | **1153** | | **-** | | | **-** |
| 142 | 700 | 0.640 | | Dec-1-ene | | | | 872-05-9 | | | C10H20 | 1191 | | 1191 | | | [33] |
| 143 | 710 | 0.590 | | Dodecane | | | | 112-40-3 | | | C12H26 | 1197 | | 1201 | | | [1] |
| 144 | 870 | 0.610 | | Tridecane | | | | 629-50-5 | | | C13H28 | 1297 | | 1301 | | | [1] |
| 145 | 895 | 0.580 | | Isocetane | | | | 4390-04-9 | | | C16H34 | 1314 | | 1317 | | | [34] |
| 146 | 1020 | 3.370 | | Tetradecane | | | | 629-59-4 | | | C14H30 | 1402 | | 1401 | | | [1] |
| 147 | 1155 | 0.690 | | Pentadec-1-ene | | | | 13360-61-7 | | | C15H30 | 1497 | | 1492 | | | [35] |
| 148 | 1165 | 0.640 | | Pentadecane | | | | 629-62-9 | | | C15H32 | 1504 | | 1501 | | | [1] |
| 149 | 1300 | 0.640 | | Hexadecane | | | | 544-76-3 | | | C16H34 | 1601 | | 1601 | | | [1] |
| **150** | **1425** | **0.630** | | **Heptadecane** | | | | **629-78-7** | | | **C17H36** | **1701** | | **1701** | | | [1] |
| 151 | 1530 | 0.630 | | Octadecane | | | | 593-45-3 | | | C18H38 | 1801 | | 1754 | | | [1] |
| 152 | 1620 | 0.630 | | Nonadecane | | | | 629-92-5 | | | C19H40 | 1895 | | 1919 | | | [1] |
| 153 | 1710 | 0.640 | | Eicosane | | | | 112-95-8 | | | C20H42 | 2001 | | **-** | | | **-** |
| 154 | 1785 | 0.650 | | Heneicosane | | | | 629-94-7 | | | C21H44 | 2089 | | **-** | | | **-** |
|  |  |  | |  | | | |  | | |  |  | |  | | |  |
| *Cyclic* | | | | | | | | | | | | | | | | | |
| 155 | 250 | 4.290 | | Cyclooctatetraene | | | | 629-20-9 | | | C8H8 | 899 | | 895 | | | [1] |
| 156 | 450 | 3.330 | | (1,2-Dimethylpropyl)cyclopropane | | | | 6976-27-8 | | | C8H16 | 1040 | | **-** | | | **-** |
|  |  |  | |  | | | |  | | |  |  | |  | | |  |
| *Aromatic* | | | | | | | | | | | | | | | | | |
| 157 | 95 | 0.630 | | Benzene | | | | 71-43-2 | | | C6H6 | 654 | | 643 | | | [1] |
| 158 | 140 | 0.810 | | Toluene | | | | 108-88-3 | | | C7H8 | 767 | | 759 | | | [1] |
| 159 | 215 | 0.980 | | Ethylbenzene | | | | 100-41-4 | | | C8H10 | 861 | | 860 | | | [1] |
| **160** | **225** | **1.020** | | **1,3-Dimethylbenzene** | | | | **108-38-3** | | | **C8H10** | **871** | | **871** | | | [1] |
| 161 | 245 | 3.780 | | 1,2-Dimethylbenzene | | | | 95-47-6 | | | C8H10 | 894 | | 901 | | | [1] |
| 162 | 250 | 1.640 | | Styrene | | | | 100-42-5 | | | C8H8 | 897 | | 895 | | | [36] |
| **163** | **335** | **3.850** | | **1-Ethyl-2-methylbenzene** | | | | **611-14-3** | | | **C9H12** | **962** | | **973** | | | [37] |
| **164** | **340** | **1.120** | | **1-Methylethylbenzene** | | | | **98-82-8** | | | **C9H12** | **964** | | **927** | | | [1] |
| 165 | 345 | 1.200 | | 1,3,5-Trimethylbenzene | | | | 108-67-8 | | | C9H12 | 968 | | 995 | | | [1] |
| 166 | 370 | 1.660 | | (1-Methylethenyl)benzene | | | | 98-83-9 | | | C9H10 | 986 | | 985 | | | [1] |
| 167 | 385 | 1.280 | | 1,2,3-Trimethylbenzene | | | | 526-73-8 | | | C9H12 | 997 | | 1023 | | | [1] |
| **168** | **515** | **1.270** | | **1-Ethyl-2,3-dimethylbenzene** | | | | **933-98-2** | | | **C10H14** | **1079** | | **1079** | | | [1] |
| **169** | **685** | **3.760** | | **Naphthalene** | | | | **91-20-3** | | | **C10H8** | **1184** | | **1189** | | | [38] |
| 170 | 800 | 0.890 | | 1,3-bis(1,1-Dimethylethyl)benzene | | | | 1014-60-4 | | | C14H22 | 1254 | | **-** | | | **-** |
| 171 | 885 | 3.540 | | 1-Methylnaphthalene | | | | 90-12-0 | | | C11H10 | 1309 | | 1307 | | | [37] |
| **172** | **915** | **1.940** | | **4-Phenylcyclohexene** | | | | **4994-16-5** | | | **C12H14** | **1329** | | **1345** | | | [39] |
| **173** | **990** | **3.720** | | **Biphenyl** | | | | **92-52-4** | | | **C12H10** | **1382** | | **1383** | | | [1] |
| 174 | 1360 | 0.950 | | 1-Propylnonylbenzene | | | | 2719-64-4 | | | C18H30 | 1649 | | 1648 | | | [1] |
| **175** | **1370** | **2.550** | | **1,1'-(1,3-Propanediyl)bis-benzene** | | | | **1081-75-0** | | | **C15H16** | **1658** | | **1654** | | | [1] |
| **176** | **1405** | **2.260** | | **1,1'-(1-Methyl-1,3-propanediyl)bis-benzene** | | | | **1520-44-1** | | | **C16H18** | **1687** | | **1664** | | | [40] |
| 177 | 1425 | 2.410 | | 1,1'-(1-Butenylidene)bis-benzene | | | | 1726-14-3 | | | C16H16 | 1702 | | - | | | - |
| 178 | 1445 | 1.800 | | 1-Phenyl-1,3,3-trimethylindan | | | | 3910-35-8 | | | C18H20 | 1721 | | 1716 | | | [41] |
| 179 | 1450 | 1.900 | | 2,6-Diisopropylnaphthalene | | | | 24157-81-1 | | | C16H20 | 1726 | | 1728 | | | [37] |
| 180 | 1630 | 0.950 | | (1-Methyldodecyl)benzene | | | | 4534-53-6 | | | C19H32 | 1907 | | 1916 | | | [29] |
|  |  |  | |  | | | |  | | |  |  | |  | | |  |
|  | | | | | | | | | | | | | | | | | |
| ***Ketones* (17.8%)** | | | | | | | | | | | | | | | | | |
| *Aliphatic* | | | | | | | | | | | | | | | | | |
| 181 | 60 | 3.080 | | Propan-2-one | | | | 67-64-1 | | | C3H6O | 584 | | 559 | | | [1] |
| 182 | 80 | 0.600 | | Butan-2-one | | | | 78-93-3 | | | C4H8O | 625 | | 622 | | | [42] |
| 183 | 80 | 0.760 | | Butane-2,3-dione | | | | 431-03-8 | | | C4H6O2 | 625 | | 591 | | | [1] |
| **184** | **100** | **0.720** | | **Pentan-2-one** | | | | **107-87-9** | | | **C5H10O** | **672** | | **664** | | | [1] |
| 185 | 105 | 0.970 | | Pentane-2,3-dione | | | | 600-14-6 | | | C5H8O2 | 679 | | 665 | | | [1] |
| 186 | 110 | 3.040 | | Acetoin | | | | 513-86-0 | | | C4H8O2 | 701 | | 698 | | | [1] |
| **187** | **115** | **3.470** | | **4-Methylpentan-2-one** | | | | **108-10-1** | | | **C6H12O** | **714** | | **736** | | | [43] |
| 188 | 150 | 0.850 | | Hexan-3-one | | | | 589-38-8 | | | C6H12O | 790 | | 795 | | | [44] |
| **189** | **155** | **0.940** | | **Hexan-2-one** | | | | **591-78-6** | | | **C6H12O** | **801** | | **790** | | | [45] |
| **190** | **160** | **1.160** | | **4-Methylpent-3-en-2-one** | | | | **141-79-7** | | | **C6H10O** | **806** | | **801** | | | [1] |
| **191** | **190** | **0.870** | | **5-Methylhexan-3-one** | | | | **623-56-3** | | | **C7H14O** | **836** | | **865** | | | [46] |
| 192 | 215 | 1.060 | | 5-Methylhexan-2-one | | | | 110-12-3 | | | C7H14O | 861 | | 860 | | | [1] |
| 193 | 230 | 0.970 | | Heptan-4-one | | | | 123-19-3 | | | C7H14O | 876 | | 871 | | | [1] |
| 194 | 245 | 1.050 | | Heptan-3-one | | | | 106-35-4 | | | C7H14O | 891 | | 889 | | | [1] |
| 195 | 250 | 1.170 | | Heptan-2-one | | | | 110-43-0 | | | C7H14O | 896 | | 895 | | | [1] |
| **196** | **310** | **1.130** | | **4-Methylheptan-2-one** | | | | **6137-06-0** | | | **C8H16O** | **942** | | **943** | | | [47] |
| 197 | 330 | 1.220 | | 6-Methylheptan-2-one | | | | 928-68-7 | | | C8H16O | 956 | | 956 | | | [30] |
| **198** | **335** | **1.310** | | **4-Methylhept-3-en-2-one** | | | | **22319-25-1** | | | **C8H14O** | **960** | | **-** | | | **-** |
| **199** | **345** | **1.270** | | **5-Methylheptan-2-one** | | | | **18217-12-4** | | | **C8H16O** | **968** | | **971** | | | [48] |
| 200 | 380 | 1.660 | | 6-Methylhept-5-en-2-one | | | | 110-93-0 | | | C8H14O | 993 | | 985 | | | [1] |
| 201 | 380 | 1.160 | | Octan-3-one | | | | 106-68-3 | | | C8H16O | 993 | | 990 | | | [1] |
| 202 | 385 | 1.300 | | Octan-2-one | | | | 111-13-7 | | | C8H16O | 997 | | 990 | | | [1] |
| **203** | **460** | **1.830** | | **Oct-3-en-2-one** | | | | **1669-44-9** | | | **C8H14O** | **1045** | | **1040** | | | [1] |
| **204** | **485** | **1.300** | | **7-Methyloctan-3-one** | | | | **5408-57-1** | | | **C9H18O** | **1060** | | **1057** | | | [49] |
| **205** | **535** | **1.210** | | **Nonan-3-one** | | | | **925-78-0** | | | **C9H18O** | **1091** | | **1090** | | | [1] |
| 206 | 545 | 1.340 | | Nonan-2-one | | | | 821-55-6 | | | C9H18O | 1098 | | 1095 | | | [1] |
| **207** | **570** | **3.080** | | **6-Methylhepta-3,5-diene-2-one** | | | | **1604-28-0** | | | **C8H12O** | **1114** | | **1112** | | | [1] |
| 208 | 575 | 1.920 | | Phorone | | | | 504-20-1 | | | C9H14O | 1116 | | - | | | - |
| 209 | 605 | 2.240 | | 4-Acetyl-1-methylcyclohexene | | | | 6090-09-1 | | | C9H14O | 1135 | | 1131 | | | [10] |
| **210** | **700** | **1.210** | | **Decan-3-one** | | | | **928-80-3** | | | **C10H20O** | **1192** | | **1190** | | | [1] |
| 211 | 705 | 1.350 | | Decan-2-one | | | | 693-54-9 | | | C10H20O | 1195 | | 1195 | | | [1] |
| 212 | 865 | 1.330 | | Undecan-2-one | | | | 112-12-9 | | | C11H22O | 1295 | | 1291 | | | [50] |
| **213** | **985** | **1.140** | | **2-Methylundecan-5-one** | | | | **50639-02-6** | | | **C12H24O** | **1377** | | **1370** | | | [1] |
| 214 | 1010 | 1.190 | | Dodecan-3-one | | | | 1534-27-6 | | | C12H24O | 1394 | | 1396 | | | [51] |
| **215** | **1020** | **1.300** | | **Dodecan-2-one** | | | | **6175-49-1** | | | **C12H24O** | **1400** | | **1395** | | | [1] |
| **216** | **1155** | **1.200** | | **Tridecan-3-one** | | | | **1534-26-5** | | | **C13H26O** | **1497** | | **1498** | | | [52] |
| **217** | **1165** | **1.290** | | **Tridecan-2-one** | | | | **593-08-8** | | | **C13H26O** | **1505** | | **1496** | | | [53] |
| 218 | 1425 | 1.190 | | Pentadecan-2-one | | | | 2345-28-0 | | | C15H30O | 1701 | | 1698 | | | [54] |
|  |  |  | |  | | | |  | | |  |  | |  | | |  |
| *Aromatic* | | | | | | | | | | | | | | | | | |
| 219 | 505 | 4.940 | | Acetophenone | | | | 98-86-2 | | | C8H8O | 1075 | | 1074 | | | [1] |
| 220 | 605 | 4.960 | | 1-Phenylpropan-2-one | | | | 103-79-7 | | | C9H10O | 1136 | | 1135 | | | [1] |
| 221 | 695 | 4.390 | | 1-(4-Methylphenyl)ethanone | | | | 122-00-9 | | | C9H10O | 1191 | | 1179 | | | [1] |
| **222** | **805** | **3.278** | | **1-Phenylbutan-1-one** | | | | **495-40-9** | | | **C10H12O** | **1258** | | **1254** | | | [1] |
| **223** | **905** | **3.060** | | **4-Isopropylacetophenone** | | | | **645-13-6** | | | **C11H14O** | **1323** | | **1320** | | | [1] |
| 224 | 1100 | 2.980 | | 1-(4-*tert*-Butylphenyl)propan-2-one | | | | 81561-77-5 | | | C13H18O | 1459 | | - | | | - |
| 225 | 1520 | 2.010 | | 1,5,6,7-Tetramethyl-3-phenylbicyclo[3.2.0]hepta-2,6-diene | | | | - | | | C17H20 | 1792 | | - | | | - |
| 226 | 1565 | 2.010 | | 2,4-Diphenyl-4-methyl-2(E)-pentene | | | | 22768-22-5 | | | C18H20 | 1839 | | 1836 | | | [10] |
|  |  |  | |  | | | |  | | |  |  | |  | | |  |
| *Cyclic* | | | | | | | | | | | | | | | | | |
| **227** | **250** | **1.900** | | **Cyclohexanone** | | | | **108-94-1** | | | **C6H10O** | **897** | | **895** | | | [1] |
| 228 | 295 | 1.550 | | 1-Cyclopentylethanone | | | | 6004-60-0 | | | C7H12O | 931 | | 932 | | | [1] |
| 229 | 560 | 3.380 | | 3,5-Dimethylcyclohex-2-en-1-one | | | | 1123-09-7 | | | C8H12O | 1108 | | - | | | - |
| 230 | 585 | 2.820 | | Isophorone | | | | 78-59-1 | | | C9H14O | 1123 | | 1124 | | | [55] |
| 231 | 615 | 1.680 | | 2-Hexylcyclopentanone | | | | 13074-65-2 | | | C11H20O | 1141 | | 1140 | | | [1] |
| 232 | 650 | 1.870 | | 3-Butylcyclopentanone | | | | 57283-81-5 | | | C9H16O | 1162 | | 1162 | | | [1] |
| 233 | 815 | 1.830 | | 3,3,5-Trimethylcyclohexanone | | | | 873-94-9 | | | C9H16O | 1264 | | 1266 | | | [1] |
| **234** | **1120** | **1.500** | | **2,6-bis(1,1-Dimethylethyl)cyclohexa-2,5-diene-1,4-dione** | | | | **719-22-2** | | | **C14H20O2** | **1473** | | **1472** | | | [56] |
| 235 | 1135 | 3.780 | | 2,6-Di(*t*-butyl)-4-hydroxy-4-methylcyclohexa-2,5-dien-1-one | | | | 10396-80-2 | | | C15H24O2 | 1485 | | 1478 | | | [57] |
| 236 | 1165 | 1.800 | | Cashmeran | | | | 33704-61-9 | | | C14H22O | 1505 | | 1503 | | | [10] |
| 237 | 1645 | 3.500 | | 7,9-Di-*tert*-butyl-1-oxaspiro(4,5)deca-6,9-diene-2,8-dione | | | | 82304-66-3 | | | C17H24O3 | 1928 | | 1929 | | | [58] |
|  |  |  | |  | | | |  | | |  |  | |  | | |  |
| ***N-compounds* (5.3%)** | | | | | | | | | | | | | | | | | |
| 238 | 125 | 1.920 | | Pyrazine | | | | 290-37-9 | | | C4H4N2 | 734 | | 734 | | | [14] |
| **239** | **135** | **1.620** | | **Pyridine** | | | | **110-86-1** | | | **C5H5N** | **757** | | **760** | | | [1] |
| 240 | 180 | 1.690 | | 2-Methylpyridine | | | | 109-06-8 | | | C6H7N | 827 | | 821 | | | [14] |
| 241 | 390 | 1.060 | | Benzonitrile | | | | 100-47-0 | | | C7H5N | 1001 | | 991 | | | [1] |
| **242** | **425** | **4.160** | | **4-Cyanocyclohexene** | | | | **100-45-8** | | | **C7H9N** | **1025** | | **-** | | | **-** |
| 243 | 465 | 0.720 | | 3-Methyl-N-(3-methylbutylidene)-1-butanamine | | | | 35448-31-8 | | | C10H21N | 1047 | | 1047 | | | [51] |
| 244 | 470 | 2.680 | | 1-Nitrohexane | | | | 646-14-0 | | | C6H13NO2 | 1052 | | 1051 | | | [1] |
| **245** | **485** | **1.530** | | **1-Isoamylpyrrole** | | | | **-** | | | **C9H15N** | **1060** | | **-** | | | **-** |
| 246 | 555 | 0.710 | | 5-Methyl-5-nitrosohexan-2-one | | | | 129354-55-8 | | | C7H13NO2 | 1104 | | - | | | - |
| 247 | 820 | 2.320 | | N-Nitrosodibutylamine | | | | 924-16-3 | | | C8H18N2O | 1267 | | - | | | - |
| 248 | 885 | 2.420 | | N,N-Dibutylformamide | | | | 761-65-9 | | | C9H19NO | 1309 | | 1307 | | | [1] |
| **249** | **990** | **1.780** | | **N-Benzylidenepropylamine** | | | | **6852-55-7** | | | **C10H13N** | **1381** | | **-** | | | **-** |
| 250 | 1130 | 0.800 | | 4-Methylpiperidine | | | | 626-58-4 | | | C6H13N | 1479 | | **-** | | | **-** |
| 251 | 1185 | 0.750 | | N,N-Dimethyltetradecylamine | | | | 112-75-4 | | | C16H35N | 1518 | | - | | | - |
| 252 | 1410 | 1.500 | | 2,4-di-*t*-Butyl-6-nitrophenol | | | | 20039-94-5 | | | C14H21NO3 | 1689 | | 1689 | | | [1] |
| 253 | 1530 | 3.560 | | N-Benzylidenephenethylamine | | | | 3240-95-7 | | | C15H15N | 1804 | | - | | | - |
| 254 | 1610 | 1.030 | | 6-Undecylamine | | | | 33788-00-0 | | | C11H25N | 1885 | | **-** | | | **-** |
|  |  |  | |  | | | |  | | |  |  | |  | | |  |
| ***S-compounds* (3.1%)** | | | | | | | | | | | | | | | | | |
| **255** | **100** | **3.480** | | **Methyl thiolacetate** | | | | **1534-08-3** | | | **C3H6OS** | **679** | | **688** | | | [59] |
| 256 | 125 | 2.360 | | Thiazole | | | | 288-47-1 | | | C3H3NS | 735 | | 735 | | | [14] |
| 257 | 125 | 0.960 | | Dimethyldisulfide | | | | 624-92-0 | | | C2H6S2 | 732 | | 728 | | | [1] |
| 258 | 170 | 1.912 | | 2-Methylthiazole | | | | 3581-87-1 | | | C4H5NS | 817 | | 813 | | | [1] |
| **259** | **275** | **4.370** | | **3-(Methylthio)propanal** | | | | **3268-49-3** | | | **C4H8OS** | **918** | | **912** | | | [1] |
| **260** | **310** | **1.200** | | **S-Methyl 3-methylbutanethioate** | | | | **23747-45-7** | | | **C6H12OS** | **942** | | **938** | | | [60] |
| 261 | 345 | 2.170 | | Trisulfidedimethyl | | | | 3658-80-8 | | | C2H6S3 | 968 | | 969 | | | [1] |
| **262** | **440** | **1.460** | | **2-Acetylthiazole** | | | | **24295-03-2** | | | **C5H5NOS** | **1032** | | **1024** | | | [1] |
| 263 | 765 | 2.340 | | Benzothiazole | | | | 95-16-9 | | | C7H5NS | 1233 | | 1232 | | | [1] |
| **264** | **790** | **1.410** | | **2-Tertiobutylthiophene** | | | | **-** | | | **C8H12S** | **1248** | | **1248** | | | [1] |
|  |  |  | |  | | | |  | | |  |  | |  | | |  |
| ***Terpenic compounds* (13.8%)** | | | | | | | | | | | | | | | | | |
| *Monoterpenic compounds* | | | | | | | | | | | | | | | | | |
| *Hydrocarbon-type* | | | | | | | | | | | | | | | | | |
| **265** | **290** | **3.210** | | \| **α-Pinene** \| \| --- \| | | | | **80-56-8** | | | **C10H16** | **928** | | **938** | | | [1] |
| **266** | **325** | **0.790** | | **Verbenene** | | | | **4080-46-0** | | | **C10H14** | **952** | | **958** | | | [1] |
| 267 | 430 | 1.100 | | *p*-Cymene | | | | 99-87-6 | | | C10H14 | 1026 | | 1021 | | | [61] |
| 268 | 435 | 0.850 | | Limonene | | | | 138-86-3 | | | C10H16 | 1029 | | 1028 | | | [1] |
| 269 | 945 | 0.780 | | α-Copaene | | | | 3856-25-5 | | | C15H24 | 1349 | | 1352 | | | [62] |
|  |  |  | |  | | | |  | | |  |  | |  | | |  |
| *Oxygen-containing compounds* | | | | | | | | | | | | | | | | | |
| **270** | **440** | **0.880** | | **Eucalyptol** | | | | **470-82-6** | | | **C10H18O** | **1032** | | **1034** | | | [1] |
| 271 | 510 | 1.820 | | Linalool oxide | | | | 5989-33-3 | | | C10H18O2 | 1076 | | 1078 | | | [63] |
| 272 | 515 | 2.050 | | Dihydromircenol | | | | 18479-58-8 | | | C10H20O | 1079 | | 1073 | | | [1] |
| 273 | 530 | 1.440 | | Fenchone | | | | 126-21-6 | | | C10H16O | 1088 | | 1087 | | | [18] |
| 274 | 555 | 1.530 | | 3,7-Dimethyloctan-3-ol | | | | 78-69-3 | | | C10H22O | 1104 | | 1101 | | | [1] |
| 275 | 560 | 2.570 | | Linalool | | | | 78-70-6 | | | C10H18O | 1108 | | 1107 | | | [1] |
| **276** | **575** | **2.780** | | **Fenchol** | | | | **1632-73-1** | | | **C10H18O** | **1117** | | **1119** | | | [64] |
| **277** | **615** | **3.360** | | **Pinocarveol** | | | | **547-61-5** | | | **C10H16O** | **1141** | | **1146** | | | [1] |
| 278 | 620 | 2.210 | | Dihydroterpineol | | | | 58985-02-7 | | | C10H20O | 1144 | | 1142 | | | [65] |
| 279 | 620 | 1.820 | | Camphor | | | | 464-48-2 | | | C10H16O | 1144 | | 1143 | | | [50] |
| **280** | **625** | **2.500** | | **Camphenilanol** | | | | **465-31-6** | | | **C10H18O** | **1147** | | **1148** | | | [50] |
| 281 | 630 | 3.620 | | Verbenol | | | | 473-67-6 | | | C10H16O | 1151 | | 1151 | | | [1] |
| 282 | 635 | 1.430 | | Menthone | | | | 89-80-5 | | | C10H18O | 1152 | | 1156 | | | [1] |
| 283 | 645 | 1.710 | | Pinocamphone | | | | 18358-53-7 | | | C10H16O | 1159 | | 1160 | | | [66] |
| **284** | **650** | **2.060** | | **Pinocarvone** | | | | **30460-92-5** | | | **C10H14O** | **1162** | | **1164** | | | [10] |
| **285** | **660** | **3.550** | | **Borneol** | | | | **507-70-0** | | | **C10H18O** | **1169** | | **1174** | | | [1] |
| **286** | **670** | **2.670** | | **Levomenthol (isomer)** | | | | **2216-51-5** | | | **C10H20O** | **1174** | | **1178** | | | [67] |
| **287** | **670** | **3.200** | | **Lavandulol** | | | | **58461-27-1** | | | **C10H18O** | **1175** | | **1170** | | | [68] |
| **288** | **675** | **2.230** | | **Terpinen-4-ol** | | | | **562-74-3** | | | **C10H18O** | **1177** | | **1177** | | | [50] |
| 289 | 700 | 3.130 | | α-Terpineol | | | | 98-55-5 | | | C10H18O | 1193 | | 1196 | | | [1] |
| **290** | **705** | **1.420** | | **Cymen-8-ol** | | | | **1197-01-9** | | | **C10H14O** | **1195** | | **1191** | | | [1] |
| **291** | **705** | **2.250** | | **Myrtenal** | | | | **564-94-3** | | | **C10H14O** | **1195** | | **1201** | | | [1] |
| **292** | **715** | **4.490** | | **Myrtenol** | | | | **515-00-4** | | | **C10H16O** | **1203** | | **1191** | | | [50] |
| **293** | **730** | **2.870** | | **Verbenone** | | | | **80-57-9** | | | **C10H14O** | **1211** | | **1207** | | | [62] |
| **294** | **750** | **4.910** | | **Carveol** | | | | **99-48-9** | | | **C10H16O** | **1225** | | **1217** | | | [69] |
| 295 | 755 | 3.120 | | Cumaldehyde | | | | 122-03-2 | | | C10H12O | 1227 | | 1226 | | | [70] |
| **296** | **770** | **3.954** | | **Geraniol** | | | | **106-24-1** | | | **C10H18O** | **1237** | | **1267** | | | [63] |
| 297 | 775 | 1.990 | | Pulegone | | | | 89-82-7 | | | C10H16O | 1239 | | 1244 | | | [71] |
| **298** | **775** | **3.370** | | **Citronellol** | | | | **106-22-9** | | | **C10H20O** | **1240** | | **1245** | | | [63] |
| **299** | **785** | **2.730** | | **Carvone** | | | | **99-49-0** | | | **C10H14O** | **1246** | | **1246** | | | [72] |
| **300** | **790** | **2.170** | | **Carvotanacetone** | | | | **499-71-8** | | | **C10H16O** | **1248** | | **1256** | | | [73] |
| 301 | 800 | 2.530 | | Piperitone | | | | 89-81-6 | | | C10H16O | 1255 | | 1252 | | | [50] |
| **302** | **810** | **1.290** | | **Linalyl acetate** | | | | **115-95-7** | | | **C12H20O2** | **1260** | | **1254** | | | [1] |
| 303 | 850 | 1.320 | | Endobornyl Acetate | | | | 76-49-3 | | | C12H20O2 | 1285 | | 1289 | | | [1] |
| 304 | 920 | 1.440 | | Dihydrocarvyl acetate | | | | 20777-49-5 | | | C12H20O2 | 1332 | | 1344 | | | [74] |
| **305** | **950** | **1.490** | | **β-Terpenyl acetate** | | | | **10198-23-9** | | | **C12H20O2** | **1353** | | **1351** | | | [1] |
| 306 | 985 | 1.440 | | Isobornyl acrylate | | | | 5888-35-5 | | | C13H20O2 | 1377 | | - | | | - |
| 307 | 1010 | 1010 | | Geraniol acetate | | | | 105-87-3 | | | C12H20O2 | 1401 | | 1382 | | | [1] |
| **308** | **1105** | **1105** | | **Geranylacetone** | | | | **3796-70-1** | | | **C13H22O** | **1462** | | **1455** | | | [53] |
|  |  |  | |  | | | |  | | |  |  | |  | | |  |
| ***Sesquiterpenes* (2.8%)** | | | | | | | | | | | | | | | | | |
| *Hydrocarbon-type* | | | | | | | | | | | | | | | | | |
| 309 | 1020 | 0.930 | | Longifolene | | | | 475-20-7 | | | C15H24 | 1401 | | 1413 | | | [1] |
| **310** | **1160** | **1.630** | | **α-Muurolene** | | | | **10208-80-7** | | | **C15H24** | **1501** | | **1499** | | | [50] |
| 311 | 1200 | 1.280 | | Calamenene | | | | 483-77-2 | | | C15H22 | 1530 | | 1526 | | | [75] |
| **312** | **1200** | **1.050** | | **δ-Cadinene** | | | | **483-76-1** | | | **C15H24** | **1529** | | **1530** | | | [1] |
| 313 | 1225 | 1.520 | | α-Calacorene | | | | 21391-99-1 | | | C15H20 | 1548 | | 1548 | | | [1] |
|  |  |  | |  | | | |  | | |  |  | |  | | |  |
| *Oxygen-containing compounds* | | | | | | | | | | | | | | | | | |
| **314** | **1275** | **1.660** | | **Caryophyllene oxide** | | | | **1139-30-6** | | | **C15H24O** | **1583** | | **1578** | | | [31] |
| 315 | 1300 | 2.260 | | Cedrol | | | | 77-53-2 | | | C15H26O | 1602 | | 1613 | | | [1] |
| 316 | 1340 | 1.650 | | Cubenol | | | | 21284-22-0 | | | C15H26O | 1633 | | 1643 | | | [62] |
| 317 | 1480 | 1.490 | | Ambrox | | | | 65588-69-4 | | | C15H26O | 1754 | | 1756 | | | [76] |
|  |  |  | |  | | | |  | | |  |  | |  | | |  |
| **Norisoprenoids (0.9%)** | | | | | | | | | | | | | | | | | |
| 318 | 1065 | 1.910 | | α-Ionone | | | | 127-41-3 | | | C13H20O | 1434 | | 1429 | | | [77] |
| 319 | 1140 | 1.550 | | α-Isomethylionone | | | | 127-51-5 | | | C14H22O | 1487 | | 1480 | | | [78] |
| 320 | 1150 | 2.000 | | Ionone | | | | 14901-07-6 | | | C13H20O | 1494 | | 1493 | | | [79] |

^a^ Retention times for first (^1^*t*_R_) and second (^2^*t*_R_) dimensions in seconds.

^b^ RI_Calc_: Linear Retention Index obtained through the modulated chromatogram.

^c^ RI_Lit_: Linear Retention Index reported in the literature for Equity-5 column or equivalents

**References**

1. Costa CP, Gonçalves Silva D, Rudnitskaya A, Almeida A, Rocha SM. Shedding light on *Aspergillus niger* volatile exometabolome. Sci Rep. 2016;6:27441; https://doi.org/10.1038/srep27441.

2. Pino JA, Mesa J, Muñoz Y, Martí MP, Marbot R. Volatile Components from Mango (*Mangifera indica* L.) Cultivars. J Agric Food Chem.. 2005;53:2213–2223; https://doi.org/10.1021/jf0402633.

3. Da Fonseca AM, Bizerra AMC, De Souza JSN, Monte FJQ, De Oliveira MDCF, De Mattos MC, Cordell GA, Braz-Filho R, Lemos TLG. Constituents and antioxidant activity of two varieties of coconut water (*Cocos nucifera* L.). Rev Bras Farmacogn. 2009;19:193–198; https://doi.org/10.1590/S0102-695X2009000200002.

4. Lef J, Dalrymple-Alford E. Volatile constituents of perique tobacco. Electron J Environ, Agric Food Chem. 2005;4:899–915.

5. Bogoslovsky YuN, Anvaer BI, Vigdergauz MS. Chromatographic constants in gas chromatography (in Russian). Stand Publ House. 1978;192:.

6. Goeminne PC, Vandendriessche T, Van Eldere J, Nicolai BM, Hertog MLATM, Dupont LJ. Detection of *Pseudomonas aeruginosa* in sputum headspace through volatile organic compound analysis. Respir Res. 2012;13:; https://doi.org/10.1186/1465-9921-13-87.

7. da Silva Junkes B, Amboni R, Augusto R. Semiempirical Topological Index: A Novel Molecular Descriptor for Quantitative Structure–Retention Relationship Studies. Internet Electron J Mol Des. 2003;2:33–49.

8. Dickschat JS, Martens T, Brinkhoff T, Simon M, Schulz S. Volatiles Released by a *Streptomyces* Species Isolated from the North Sea. Chem Biodivers. 2005;2:837–865; https://doi.org/10.1002/cbdv.200590062.

9. de Souza PP, Cardeal Z de L, Augusti R, Morrison P, Marriott PJ. Determination of volatile compounds in Brazilian distilled cachaça by using comprehensive two-dimensional gas chromatography and effects of production pathways. J Chromatogr A. 2009;1216:2881–2890; https://doi.org/10.1016/j.chroma.2008.10.061.

10. Andriamaharavo NR. Retention Data. NIST Mass Spectrometry Data Center. NIST Mass Spectrometry Data Center

11. Jerković I, Hegić G, Marijanović Z, Bubalo D. Organic Extractives from *Mentha* spp. Honey and the Bee-Stomach: Methyl Syringate, Vomifoliol, Terpenediol I, Hotrienol and Other Compounds. Molecules. 2010;15:2911–2924; https://doi.org/10.3390/molecules15042911.

12. Mockute D, Bernotiene G, Judzentiene Asta. Volatile compounds of the aerial parts of wild St. John’s wort (*Hypericum perforatum* L.) plants. Chemija. 2003;14:108–111.

13. Dallüge J, Van Stee LLP, Xu X, Williams J, Beens J, Vreuls RJJ, Brinkman UAT. Unravelling the composition of very complex samples by comprehensive gas chromatography coupled to time-of-flight mass spectrometry: Cigarette smoke. J Chromatogr A. 2002;974:169–184; https://doi.org/10.1016/S0021-9673(02)01384-5.

14. Methven L, Tsoukka M, Oruna-Concha MJ, Parker JK, Mottram DS. Influence of sulfur amino acids on the volatile and nonvolatile components of cooked salmon (*Salmo salar*). J Agric Food Chem. 2007;55:1427–1436; https://doi.org/10.1021/jf0625611.

15. Asuming WA, Beauchamp PS, Descalzo JT, Dev BC, Dev V, Frost S, Ma CW. Essential oil composition of four *Lomatium* Raf. species and their chemotaxonomy. Biochem Syst Ecol. 2005;33:17–26; https://doi.org/10.1016/j.bse.2004.06.005.

16. Zhao Y, Li J, Xu Y, Duan H, Fan W, Zhao G. Extraction, preparation and identification of volatile compounds in Changyu XO brandy. Chin J Chromatogr (Se Pu). 2008;26:212–222; https://doi.org/10.1016/s1872-2059(08)60014-0.

17. Jarunrattanasri A, Theerakulkait C, Cadwallader KR. Aroma components of acid-hydrolyzed vegetable protein made by partial hydrolysis of rice bran protein. J Agric Food Chem. 2007;55:3044–3050; https://doi.org/10.1021/jf0631474.

18. Adams RP. The leaf essential oils and chemotaxonomy of *Juniperus* sect. *Juniperus*. Biochem Syst Ecol. 1998;26:637–645; https://doi.org/10.1016/S0305-1978(98)00020-9.

19. Janzanntti NS, Franco MRB, Lanças FM. Identificação de compostos voláteis de maçãs (*Malus domestica*) cultivar Fuji, por cromatografia gasosa-espectrometria de massas. Ciênc Tecnol Aliment. 2000;20:; https://doi.org/10.1590/s0101-20612000000200007.

20. Olson KL, Wong CA, Fleck LL, Lazar DF. Qualitative and Quantitative Determination of Solvent Formulations in Automotive Paints. J Chromatogr Sci. 1987;25:418–423; https://doi.org/10.1093/chromsci/25.9.418.

21. Guisto R, Smith SR, Stuart JD, Hubball J. Gas chromatographic retention indices, mass, and infrared spectra of industrially important adipate esters. J Chromatogr Sci. 1993;31:225–230; https://doi.org/10.1093/chromsci/31.6.225.

22. Yoo SJ, Pace G V., Khoo BK, Lech J, Hartman TG. Mass spectrometry and gas chromatographic retention indices of selected UV/EB-curable monomers and photoinitiators commonly used in food packaging print and coating formulations. Radtech Rep. 2004;18:60–68.

23. Mondello L, Costa R. A new generation of GC capillary columns: SLB-5ms. The Reporter (Europe). 2006;20:17–19.

24. Pino JA, Márquez E, Quijano CE, Castro D. Volatile compounds in noni (*Morinda citrifolia* L.) at two ripening stages. Ciênc Tecnol Aliment. 2010;30:183–187; https://doi.org/10.1590/S0101-20612010000100028.

25. El-Massry KF, El-Ghorab AH, Farouk A. Antioxidant activity and volatile components of Egyptian *Artemisia judaica* L. Food Chem. 2002;79:331–336; https://doi.org/10.1016/S0308-8146(02)00164-4.

26. Zaikin VG. Personal communication: Retention indices measured during 2010

27. Sibanda S, Chigwada G, Poole M, Gwebu ET, Noletto JA, Schmidt JM, Rea AI, Setzer WN. Composition and bioactivity of the leaf essential oil of *Heteropyxis dehniae* from Zimbabwe. J Ethnopharmacol. 2004;92:107–111; https://doi.org/10.1016/j.jep.2004.02.010.

28. Verevkin SP, Krasnykh EL, Vasiltsova T V., Heintz A. Determination of ambient temperature vapor pressures and vaporization enthalpies of branched ethers. J Chem Eng Data. 2003;48:591–599; https://doi.org/10.1021/je0255980.

29. Mondello L. HS-SPME-GCxGC-MS analysis of Yerba Mate (*Ilex paraguariensis*) in Shimadzu GC-GC application compendium of comprehensive 2D GC. Shimadzu Corp. 2012;1–5:1–25.

30. Elmore JS, Cooper SL, Enser M, Mottram DS, Sinclair LA, Wilkinson RG, Wood JD. Dietary manipulation of fatty acid composition in lamb meat and its effect on the volatile aroma compounds of grilled lamb. Meat Sci. 2005;69:233–242; https://doi.org/10.1016/j.meatsci.2004.07.002.

31. Hazzit M, Baaliouamer A, Faleiro ML, Miguel MG. Composition of the essential oils of *Thymus* and *Origanum* species from Algeria and their antioxidant and antimicrobial activities. J Agric Food Chem. 2006;54:6314–6321; https://doi.org/10.1021/jf0606104.

32. Yang Z, Yang W, Peng Q, He Q, Feng Y, Luo S, Yu Z. Volatile phytochemical composition of rhizome of ginger after extraction by headspace solid-phase microextraction, petrol ether extraction and steam distillation extraction. Bangladesh J Pharmacol. 2009;4:136–143; https://doi.org/10.3329/bjp.v4i2.3232.

33. Lai WC, Song C. Temperature-programmed retention indices for g.c. and g.c.-m.s. analysis of coal- and petroleum-derived liquid fuels. Fuel. 1995;74:1436–1451; https://doi.org/10.1016/0016-2361(95)00108-H.

34. Kotowska U, Żalikowski M, Isidorov VA. HS-SPME/GC-MS analysis of volatile and semi-volatile organic compounds emitted from municipal sewage sludge. Environ Monit Assess. 2012;184:2893–2907; https://doi.org/10.1007/s10661-011-2158-8.

35. Zeng YX, Zhao CX, Liang YZ, Yang H, Fang HZ, Yi LZ, Zeng Z Da. Comparative analysis of volatile components from *Clematis* species growing in China. Analy Chim Acta. 2007;595:328–339; https://doi.org/10.1016/j.aca.2006.12.022.

36. Lozano PR, Miracle ER, Krause AJ, Drake MA, Cadwallader KR. Effect of cold storage and packaging material on the major aroma components of sweet cream butter. J Agric Food Chem. 2007;55:7840–7846; https://doi.org/10.1021/jf071075q.

37. Song C, Lai W-C, Madhusudan Reddy and Boli Wei K, Wei B. Temperature-Programmed Retention Indices for GC and GC-MS of Hydrocarbon Fuels and Simulated Distillation GC of Heavy Oils. Anal Adv Hydrocarb Res. 2003;147–210; https://doi.org/10.1007/978-1-4419-9212-3_7.

38. Adamova M, Orinak A, Halas L. Retention indices as identification tool in pyrolysis-capillary gas chromatography. J Chromatogr a. 2005;1087:131–141.

39. Landy P, Nicklaus S, Sémon E, Mielle P, Guichard E. Representativeness of Extracts of Offset Paper Packaging and Analysis of the Main Odor-Active Compounds. J Agric Food Chem. 2004;52:2326–2334; https://doi.org/10.1021/jf0352677.

40. Shapi MM, Hesso A. Thermal decomposition of polystyrene: Volatile compounds from large-scale pyrolysis. J Anal Appl Pyrolysis. 1990;18:143–161; https://doi.org/10.1016/0165-2370(90)80004-8.

41. Boatright WL, Crum AD. Nonpolar-volatile lipids from soy protein isolates and hexane-defatted flakes. JAOCS, J Am Oil Chem Soc. 1997;74:461–467; https://doi.org/10.1007/s11746-997-0107-z.

42. Bylaite E, Meyer AS. Characterisation of volatile aroma compounds of orange juices by three dynamic and static headspace gas chromatography techniques. Eur Food Res Technol. 2006;222:176–184; https://doi.org/10.1007/s00217-005-0141-8.

43. Engel E, Ratel J. Correction of the data generated by mass spectrometry analyses of biological tissues: Application to food authentication. J Chromatogr A. 2007;1154:331–341; https://doi.org/10.1016/j.chroma.2007.02.012.

44. Boylston TD, Vinyard BT. Isolation of volatile flavor compounds from peanut butter using purge-and-trap techniques. Dev Food Sci. 1998;39:225–243; https://doi.org/10.1016/S0167-4501(98)80011-3.

45. Wu S, Zorn H, Krings U, Berger RG. Volatiles from submerged and surface-cultured beefsteak fungus, *Fistulina hepatica*. Flavour Fragr J. 2007;22:53–60; https://doi.org/10.1002/ffj.1758.

46. Guichard E, Souty M. Comparison of the relative quantities of aroma compounds found in fresh apricot (*Prunus armeniaca*) from six different varieties. Z Lebensm Unters Forsch. 1988;186:301–307; https://doi.org/10.1007/BF01027031.

47. Timón ML, Ventanas J, Martín L, Tejeda JF, García C. Volatile Compounds in Supercritical Carbon Dioxide Extracts of Iberian Ham. J Agric Food Chem. 1998;46:5143–5150; https://doi.org/10.1021/jf980652v.

48. Owens JD, Allagheny N, Kipping G, Ames JM. Formation of Volatile Compounds During *Bacillus subtilis* Fermentation of Soya Beans. J Sci Food Agric. 1997;74:132–140; https://doi.org/10.1002/(SICI)1097-0010(199705)74:1<132::AID-JSFA779>3.0.CO;2-8.

49. Dickschat JS, Wenzel SC, Bode HB, Müller R, Schulz S. Biosynthesis of Volatiles by the Myxobacterium *Myxococcus xanthus*. ChemBioChem. 2004;5:778–787; https://doi.org/10.1002/cbic.200300813.

50. Adams RP, González Elizondo MS, Elizondo MG, Slinkman E. DNA fingerprinting and terpenoid analysis of Juniperus blancoi var. *huehuentensis* (Cupressaceae), a new subalpine variety from Durango, Mexico. Biochem Syst Ecol 2006;34:205–211; https://doi.org/10.1016/j.bse.2005.11.004.

51. Dickschat JS, Bode HB, Wenzel SC, Müller R, Schulz S. Biosynthesis and identification of volatiles released by the myxobacterium *Stigmatella aurantiaca*. ChemBioChem. 2005;6:2023–2033; https://doi.org/10.1002/cbic.200500174.

52. Xu LL, Han T, Wu JZ, Zhang QY, Zhang H, Huang BK, Rahman K, Qin LP. Comparative research of chemical constituents, antifungal and antitumor properties of ether extracts of *Panax ginseng* and its endophytic fungus. Phytomedicine. 2009;16:609–616; https://doi.org/10.1016/j.phymed.2009.03.014.

53. Adams RP, Morris JA, Pandey RN, Schwarzbach AE. Cryptic speciation between *Juniperus deltoides* and *Juniperus oxycedrus* (Cupressaceae) in the Mediterranean. Biochem Syst Ecol. 2005;33:771–787; https://doi.org/10.1016/j.bse.2005.01.001.

54. Salido S, Altarejos J, Nogueras M, Sánchez A, Pannecouque C, Witvrouw M, De Clercq E. Chemical studies of essential oils of *Juniperus oxycedrus* ssp. badia. J Ethnopharmacol. 2002;81:129–134; https://doi.org/10.1016/S0378-8741(02)00045-4.

55. Alissandrakis E, Tarantilis PA, Harizanis PC, Polissiou M. Comparison of the volatile composition in thyme honeys from several origins in Greece. J Agric Food Chem. 2007;55:8152–8157; https://doi.org/10.1021/jf071442y.

56. Kallio M, Jussila M, Rissanen T, Anttila P, Hartonen K, Reissell A, Vreuls R, Adahchour M, Hyötyläinen T. Comprehensive two-dimensional gas chromatography coupled to time-of-flight mass spectrometry in the identification of organic compounds in atmospheric aerosols from coniferous forest. J Chromatogr A. 2006;1125:234–243; https://doi.org/10.1016/j.chroma.2006.05.050.

57. Ansorena D, Gimeno O, Astiasarán I, Bello J. Analysis of volatile compounds by GC-MS of a dry fermented sausage: Chorizo de Pamplona. Food Res Int. 2001;34:67–75; https://doi.org/10.1016/S0963-9969(00)00133-2.

58. Lalel HJD, Singh Z, Tan SC. Glycosidically-bound aroma volatile compounds in the skin and pulp of “Kensington Pride” mango fruit at different stages of maturity. Postharvest Biol Technol. 2003;29:205–218; https://doi.org/10.1016/S0925-5214(02)00250-8.

59. Dirinck PJ, De Footer HL, Willaert GA, Schamp NM. Flavor Quality of Cultivated Strawberries: The Role of the Sulfur Compounds. J Agric Food Chem. 1981;29:316–321; https://doi.org/10.1021/jf00104a024.

60. Beaulieu JC, Grimm CC. Identification of volatile compounds in cantaloupe at various developmental stages using solid phase microextraction. J Agric Food Chem. 2001;49:1345–1352; https://doi.org/10.1021/jf0005768.

61. Kartal N, Sokmen M, Tepe B, Daferera D, Polissiou M, Sokmen A. Investigation of the antioxidant properties of *Ferula orientalis* L. using a suitable extraction procedure. Food Chem. 2007;100:584–589; https://doi.org/10.1016/j.foodchem.2005.09.084.

62. Lucero ME, Fredrickson EL, Estell RE, Morrison AA, Richman DB. Volatile Composition of *Gutierrezia sarothrae* (Broom Snakeweed) as Determined by Steam Distillation and Solid Phase Microextraction. J Essent Oil Res. 2006;18:121–125; https://doi.org/10.1080/10412905.2006.9699039.

63. Jalali-Heravi M, Zekavat B, Sereshti H. Characterization of essential oil components of Iranian geranium oil using gas chromatography-mass spectrometry combined with chemometric resolution techniques. J Chromatogr A. 2006;1114:154–163; https://doi.org/10.1016/j.chroma.2006.02.034.

64. Sabulal B, Mathew D, Anil JJ, Rajani K, Sreeja PC, Varughese G. Phenylbutanoid-rich rhizome oil of *Zingiber neesanum* from Western Ghats, southern India. Flavour Fragr J. 2007;22:521–524; https://doi.org/10.1002/ffj.1834.

65. Ramos MFS, Siani AC, Tappin MRR, Guimarães AC, Ribeiro JELDS. Essential oils from oleoresins of *Protium* spp. of the Amazon region. Flavour Fragr J. 2000;15:383–387; https://doi.org/10.1002/1099-1026(200011/12)15:6<383::AID-FFJ927>3.0.CO;2-X.

66. Mockute D, Bernotiene G, Judzentiene A. The Essential Oil of Ground Ivy (*Glechoma hederacea* L) Growing Wild In Eastern Lithuania. J Essent Oil Res. 2007;19:449–451; https://doi.org/10.1080/10412905.2007.9699948.

67. Lorenzo D, Paz D, Dellacassa E, Davies P, Vila R, Cañigueral S. Essential oils of *Mentha pulegium* and *Mentha rotundifolia* from Uruguay. Braz Arch Biol Technol. 2002;45:519–524; https://doi.org/10.1590/S1516-89132002000600016.

68. Tret’yakov KV. Retention Data. NIST Mass Spectrometry Data Center. NIST Mass Spectrometry Data Center

69. Guilliard M, Delgado W, Martínez JR, Stashenko E. Determination of the Enantiomeric Purity of Carvone, Main Component of Colombian Lippia alba (Mill) Oil by Means of Bidimensional Gas Chromatography. 2001;1–6.

70. Gkinis G, Tzakou O, Iliopoulou D, Roussis V. Chemical Composition and Biological Activity of *Nepeta parnassica* Oils and Isolated Nepetalactones. Z Naturforsch C J Biosci. 2003;58:681–686; https://doi.org/10.1515/znc-2003-9-1015.

71. Tepe B, Sokmen M, Sokmen A, Daferera D, Polissiou M. Antimicrobial and antioxidative activity of the essential oil and various extracts of *Cyclotrichium origanifolium* (Labill.) Manden. & Scheng. J Food Eng. 2005;69:335–342; https://doi.org/10.1016/j.jfoodeng.2004.08.024.

72. Barbosa LCA, Paula VF, Azevedo AS, Silva EAM, Nascimento EA. Essential oil composition from some plant parts of *Conyza bonariensis* (L.) Cronquist. Flavour Fragr J. 2005;20:39–41; https://doi.org/10.1002/ffj.1392.

73. Gauvin A, Lecomte H, Smadja J. Comparative investigations of the essential oils of two scented geranium (*Pelargonium* spp.) cultivars grown on Reunion Island. Flavour Fragr J. 2004;19:455–460; https://doi.org/10.1002/ffj.1354.

74. Flamini G, Cioni PL, Morelli I. Volatiles from Leaves, Fruits, and Virgin Oil from *Olea europaea* Cv. Olivastra Seggianese from Italy †. J Agric Food Chem. 2003;51:1382–1386; https://doi.org/10.1021/jf020854y.

75. Cornu A, Carnat AP, Martin B, Coulon JB, Lamaison JL, Berdagué JL. Solid-phase microextraction of volatile components from natural grassland plants. J Agric Food Chem. 2001;49:203–209; https://doi.org/10.1021/jf0008341.

76. Palic R, Stojanovic G, Alagic S, Nikolic M, Lepojevic Z. Chemical composition and antimicrobial activity of the essential oil and CO2 extracts of the oriental tobacco, Prilep. Flavour Fragr J. 2002;17:323–326; https://doi.org/10.1002/ffj.1084.

77. Nickavar B, Salehi-Sormagi MH, Amin G, Daneshtalab M. Steam Volatiles of *Vaccinium arctostaphylos*. Pharm Biol. 2002;40:448–449; https://doi.org/10.1076/phbi.40.6.448.8449.

78. Mondello L, Sciarrone D, Casilli A, Tranchida PQ, Dugo P, Dugo G. Fast gas chromatography-full scan quadrupole mass spectrometry for the determination of allergens in fragrances. J Sep Sci. 2007;30:1905–1911; https://doi.org/10.1002/jssc.200600541.

79. Wang Y, Finn C, Qian MC. Impact of Growing Environment on Chickasaw Blackberry (*Rubus* L.) Aroma Evaluated by Gas Chromatography Olfactometry Dilution Analysis. J Agric Food Chem. 2005;53:3563–3571; https://doi.org/10.1021/jf048102m.
